# Progesterone increases blood glucose via hepatic progesterone receptor membrane component 1 under limited or impaired action of insulin

**DOI:** 10.1038/s41598-020-73330-7

**Published:** 2020-10-01

**Authors:** Sang R. Lee, Woo-Young Choi, Jun H. Heo, Jiyoung Huh, Globinna Kim, Kyu-Pil Lee, Hyo-Jung Kwun, Hyun-Jin Shin, In-Jeoung Baek, Eui-Ju Hong

**Affiliations:** 1 College of Veterinary Medicine, Chungnam National University, 99 Daehak-ro, Suite 401Veterinary medicine Bldg., Yuseong, Daejeon, 34134 Republic of Korea; 2 Department of Convergence Medicine, Asan Medical Center, University of Ulsan College of Medicine, Seoul, 05505 Republic of Korea

**Keywords:** Endocrinology, Gastroenterology

## Abstract

Hepatic gluconeogenesis is the main pathway for blood glucose maintenance activated during fasting. Retardation of insulin action, such as in diabetes mellitus, activates gluconeogenesis during the fed state. While the role of progesterone (P4) in diabetes is controversial, the P4 receptor, progesterone receptor membrane component 1 (PGRMC1), is known to stimulate pancreatic insulin secretion. We investigated the role of P4, via hepatic PGRMC1, during gluconeogenesis. The PGRMC1 binding chemical, AG-205, induced PGRMC1 monomer (25 kDa) abundance, and increased PEPCK expression and glucose production in parallel with cyclic AMP (cAMP) induction in Hep3B cells. PGRMC1-mediated cyclic AMP was inhibited by an adenylate cyclase inhibitor (MDL-12,330A). PEPCK suppression in Pgrmc1 KO hepatocyte was not observed after treatment of MDL-12,330A. PGRMC1 knockdown or overexpression systems in Hep3B cells confirmed that PGRMC1 mediates PEPCK expression via phosphorylation of cAMP-response element binding protein (CREB). CREB phosphorylation and PEPCK expression in primary hepatocytes were greater than that in PGRMC1 knock-out hepatocytes. Progesterone increased PGRMC1 expression, which induced cAMP and PEPCK induction and glucose production. In vivo, P4 suppressed gluconeogenesis following plasma insulin induction under normal conditions in a mouse model. However, P4 increased blood glucose via gluconeogenesis in parallel with increases in PGRMC1 and PEPCK expression in mice in both insulin-deficient and insulin-resistant conditions. We conclude that P4 increases hepatic glucose production via PGRMC1, which may exacerbate hyperglycaemia in diabetes where insulin action is limited.

## Introduction

As a part of metabolic homeostasis, our body maintains blood glucose levels within a narrow range. The liver is responsible for maintaining normal blood glucose levels^1^. In an early fasting state, insulin mainly regulates blood glucose levels by inhibiting hepatic glucose production and glycogenolysis in the liver^2^. After the stored hepatic glycogen is depleted by glycogenolysis, the body maintains a normal glucose level by promoting various hepatic processes^3^. Hepatic gluconeogenesis is the major pathway utilized during this period for the maintenance of normal plasma glucose levels by generating glucose from non-carbohydrate precursors^3,4^. As a key mediator of gluconeogenesis, the expression of phosphoenolpyruvate carboxykinase (PEPCK) is regulated by insulin and glucagon. When PEPCK expression is suppressed by insulin, it is induced by glucagon-mediated cyclic AMP (cAMP) accumulation, and performs the rate-limiting step of gluconeogenesis. As a therapeutic target for diabetes, the importance of PEPCK is therefore promising due to its involvement in gluconeogenesis^5^.

Progesterone (P4) is an essential hormone for foetal survival and pregnancy maintenance^6^. Progesterone has been discussed in previous studies in the context of gestational diabetes, but its role in gestational diabetes is controversial, as it affects various endocrine organs. According to a previous study, oestrogen and P4 in ovariectomized female mice induced the transcription of gluconeogenic genes, including *Pepck*, in the liver^7^. Conversely, P4 also promoted insulin secretion in the pancreas^8,9^. As diabetes usually involves insulin deficiency, we hypothesized that P4-mediated gluconeogenesis might increase blood glucose when insulin action is limited.

Progesterone receptor membrane component 1 (PGRMC1) is a novel cell surface receptor that is involved in cytochrome activities, drug metabolism, cholesterol synthesis, and steroid synthesis^10,11^. Recently, we found that PGRMC1 is involved in fatty liver amelioration^12^. However, it was also reported that PGRMC1 is a functional part of the GLP-1 receptor (GLP-1R) promoting insulin secretion in beta cells (β cells)^13,14^. We speculated that P4 is the causal factor for diabetes through the regulation of PGRMC1. In this study, we investigated the relationship between P4 and gluconeogenesis using an in vitro and in vivo model involving PGRMC1 and P4.

## Results

### PGRMC1 induces PEPCK and gluconeogenesis via cAMP induction by adenylate cyclase

**Figure 1:**
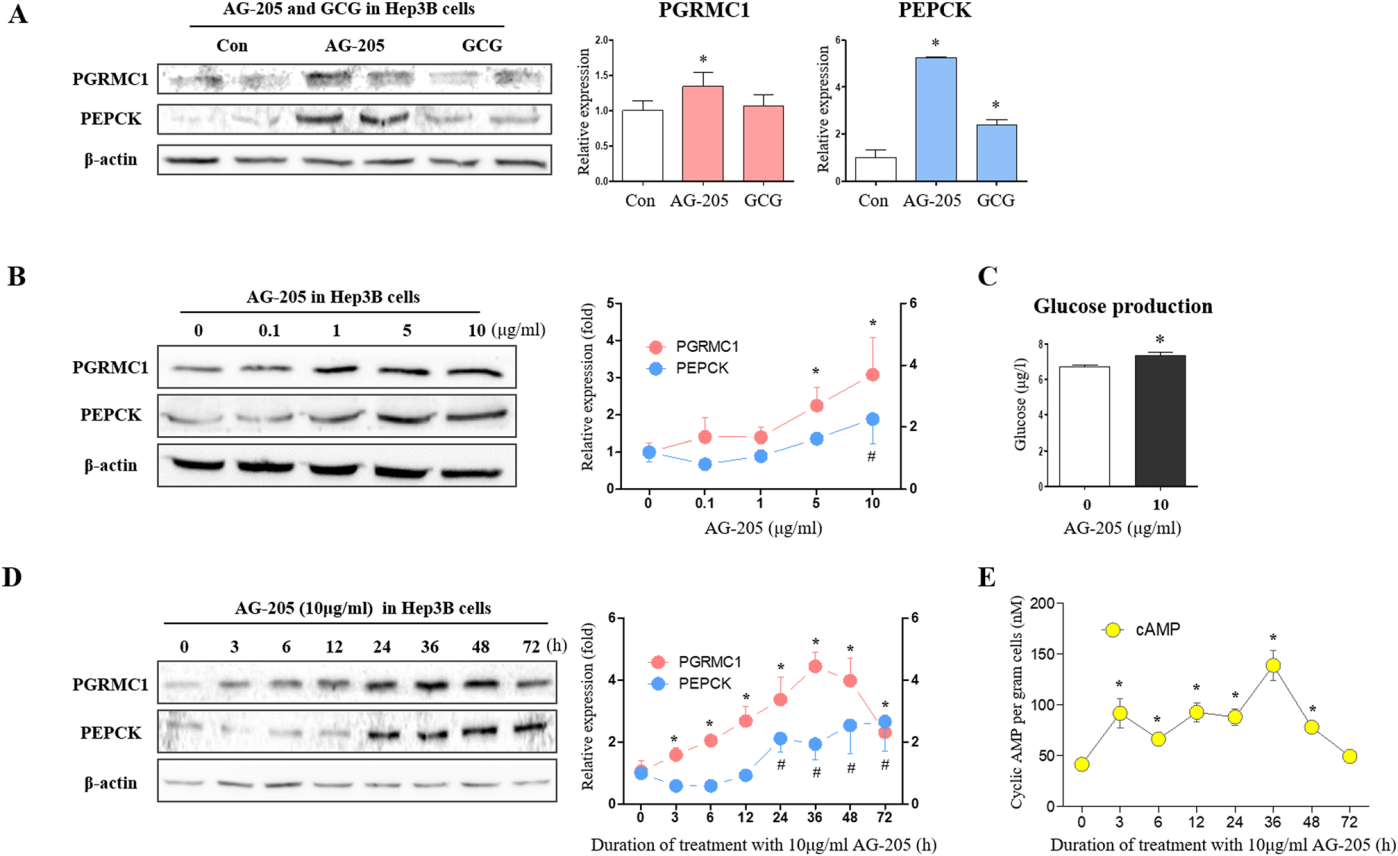
Induction of PEPCK, cyclic AMP and gluconeogenesis by AG-205. (**A**) Western blot analysis and quantification of PGRMC1 and PEPCK in Control, Glucagon (GCG), and AG-205 group. GCG (100 µM, 2 h) and AG-205 (10 µg/ml, 18 h) were treated in DMEM-low glucose medium (50 mg/dl, w/o FBS, 1% Penicillin/Streptomycin, 1 nM dexamethasone). Values represent means ± SD. *p < 0.05 versus Control. (**B**) Western blot analysis and quantification of PGRMC1 and PEPCK in control versus AG-205 group. AG-205 (0.1, 1, 5, and 10 µg/ml) was treated in DMEM-low glucose medium (50 mg/dl, w/o FBS, 1% Penicillin/Streptomycin, 1 nM dexamethasone) for 18 h. β-actin was used as an internal control. Values represent means ± SD. *p < 0.05 versus control. (**C**) Glucose production was measured by glucose peroxidase assay. After AG-205 pre-treatment (10 µg/ml) for 48 h, cells were incubated with glucose production buffer (sodium pyruvate 2 mM, dexamethasone 1 nM, w/o glucose) for 2 h. Values represent means ± SD. *p < 0.05 versus control. (**D**) Western blot analysis and quantification of PGRMC1 and PEPCK in 0 h versus 3, 6, 12, 24, 36, 48, and 72 h. AG-205 (10 µg/ml) was treated in DMEM-low glucose medium (50 mg/dl, w/o FBS, 1% Penicillin/Streptomycin, 1 nM dexamethasone) for indicated hours. β-actin was used as an internal control. Values represent means ± SD. *p < 0.05 vesus 0 h. (**E**) Cyclic AMP (cAMP) levels of indicated hours. AG-205 (10 µg/ml) was treated in DMEM-low glucose medium. Data were normalized to loading cell number, 5000 cells per well. Values represent means ± SD. *p < 0.05 versus 0 h. All experiments were repeated at least 3 times.

To investigate the relationship between PGRMC1-related gluconeogenesis and AG-205, we treated Hep3B cells cultured in low-glucose medium (50 mg/dl) with AG-205 (10 µg/ml) and used glucagon (GCG, 100 µM) treated cells as an experimental control. While GCG failed to induce PGRMC1 expression, the PGRMC1 monomer abundance was increased (*p* < 0.05, 1.35-fold vs. control) in the AG-205 group (Fig. 1A). Gluconeogenic PEPCK expression (*p* < 0.05, 5.26- and 2.39-fold, respectively vs. control) were both increased after treatment with AG-205 and GCG (Fig. 1A). Hep3B cells exposed to AG-205 in a dose-dependent manner demonstrated a significant increase in PGRMC1 expression (*p* < 0.05) (2.24-and 3.07-fold vs. control) with 5 and 10 µg/ml treatment of AG-205 (Fig. 1B). Moreover, PEPCK expression increased (*p* < 0.05, 1.88-fold vs. control) with 10 µg/mL AG-205 (Fig. 1B). In same condition, we also observed the increase of *PGRMC1* and *PEPCK* mRNA (Fig. S1). Glucose production was increased (*p* < 0.05, 1.1-fold vs. control) with 10 µg/ml AG-205 (Fig. 1C).

AG-205 treatment led to increased PGRMC1 monomer abundance (*p* < 0.05, 1.49-, 1.92-, 2.52-, 3.16-, 4.16-, 3.73-, and 2.17-fold, respectively, vs. 0 h) at 3, 6, 12, 24, 36, 48 and 72 h post-treatment (Fig. 1D). PGRMC1 expression peaked at 36 h post-treatment. PEPCK expression was increased (*p* < 0.05, 2.12-, 1.94-, 2.54- and 2.65-fold, respectively, vs. 0 h) at 24, 36, 48, and 72 h after treatment with AG-205 (Fig. 1D). These results imply that the induction of PGRMC1 occurs earlier than the induction of PEPCK expression. To investigate the reason for the time delay between increases in PGRMC1 and PEPCK levels, we monitored cellular cAMP levels at the same time points as PEPCK expression is closely related to cAMP levels during gluconeogenesis. Interestingly, levels of cAMP were increased (*p* < 0.05, 2.21-, 1.6-, 2.23-, 2.12-, 3.34-, and 1.88-fold, respectively, vs. 0 h) at 3, 6, 12, 24, 36, and 48 h post-treatment with AG-205 (Fig. 1E). The peak cAMP level was 36 h after treatment, which is similar to the timing of the PGRMC1 peak and earlier than the PEPCK peak. Therefore, cAMP levels appear to be enhanced following increases in PGRMC1 levels and may act as a secondary messenger for the induction of PEPCK expression.

**Figure 2:**
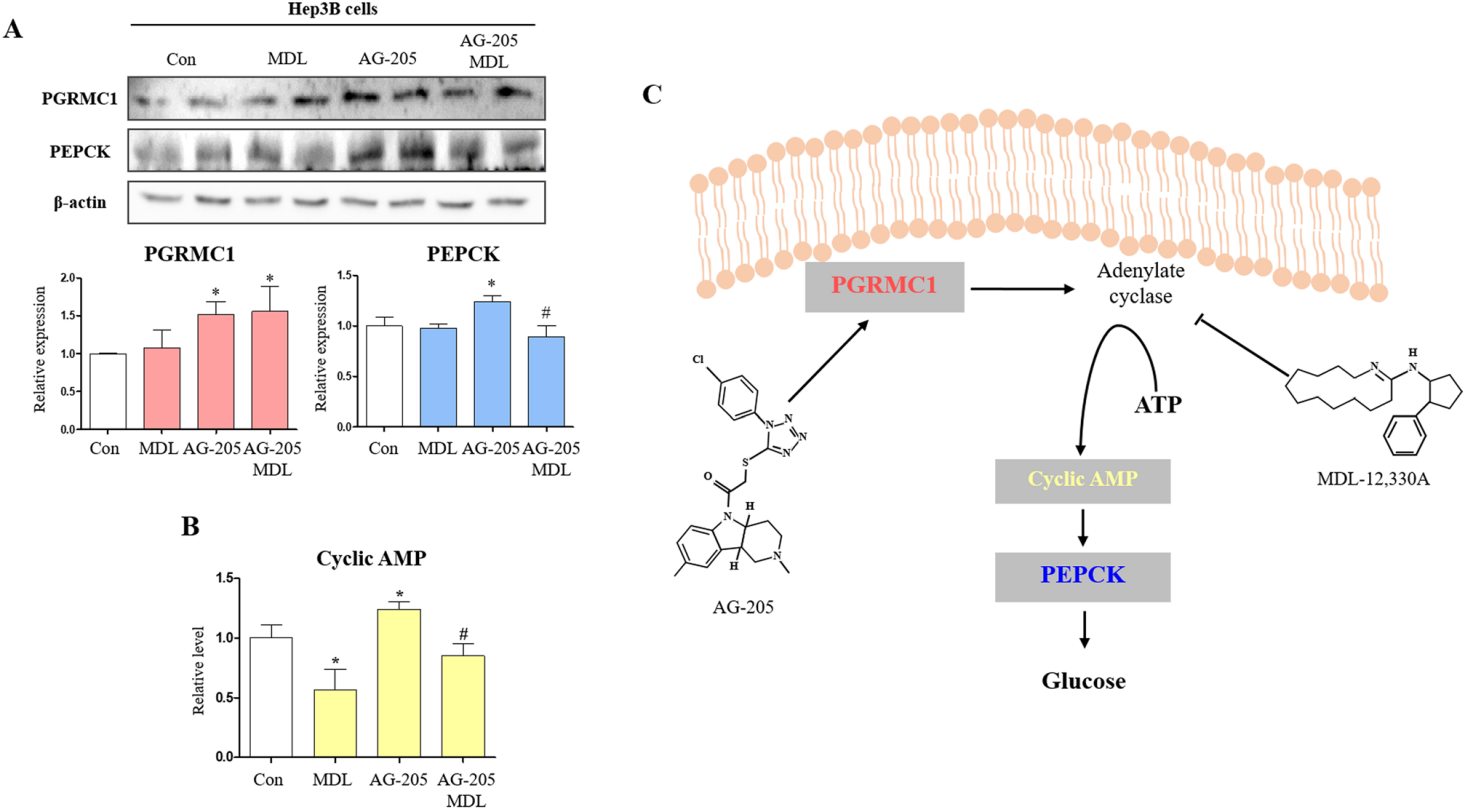
Induction of PEPCK expression by AG-205 was suppressed by adenylate cyclase inhibition. (**A**) Western blot analysis and quantification of PGRMC1 and PEPCK in Control versus MDL-12,330A, AG-205, AG-205 + MDL-12,330A group. β-actin was used as an internal control. Values represent means ± SD. *p < 0.05 versus control. #*p* < 0.05 versus AG-205 group. (**B**) Cyclic AMP (cAMP) levels after 24 h incubation in low glucose medium (50 mg/dl, w/o FBS, 1% Penicillin/Streptomycin, 100 nM dexamethasone). MDL-12,330A (MDL, 2 µM) was treated for 2 h before harvest. Data were normalized to control. Values represent means ± SD. *p < 0.05 versus control. #*p* < 0.05 versus AG-205 group. (**C**) Illustration of PGRMC1-gluconeogenesis mechanism in Hep3B cell. All experiments were repeated at least 3 times.

**Figure 3:**
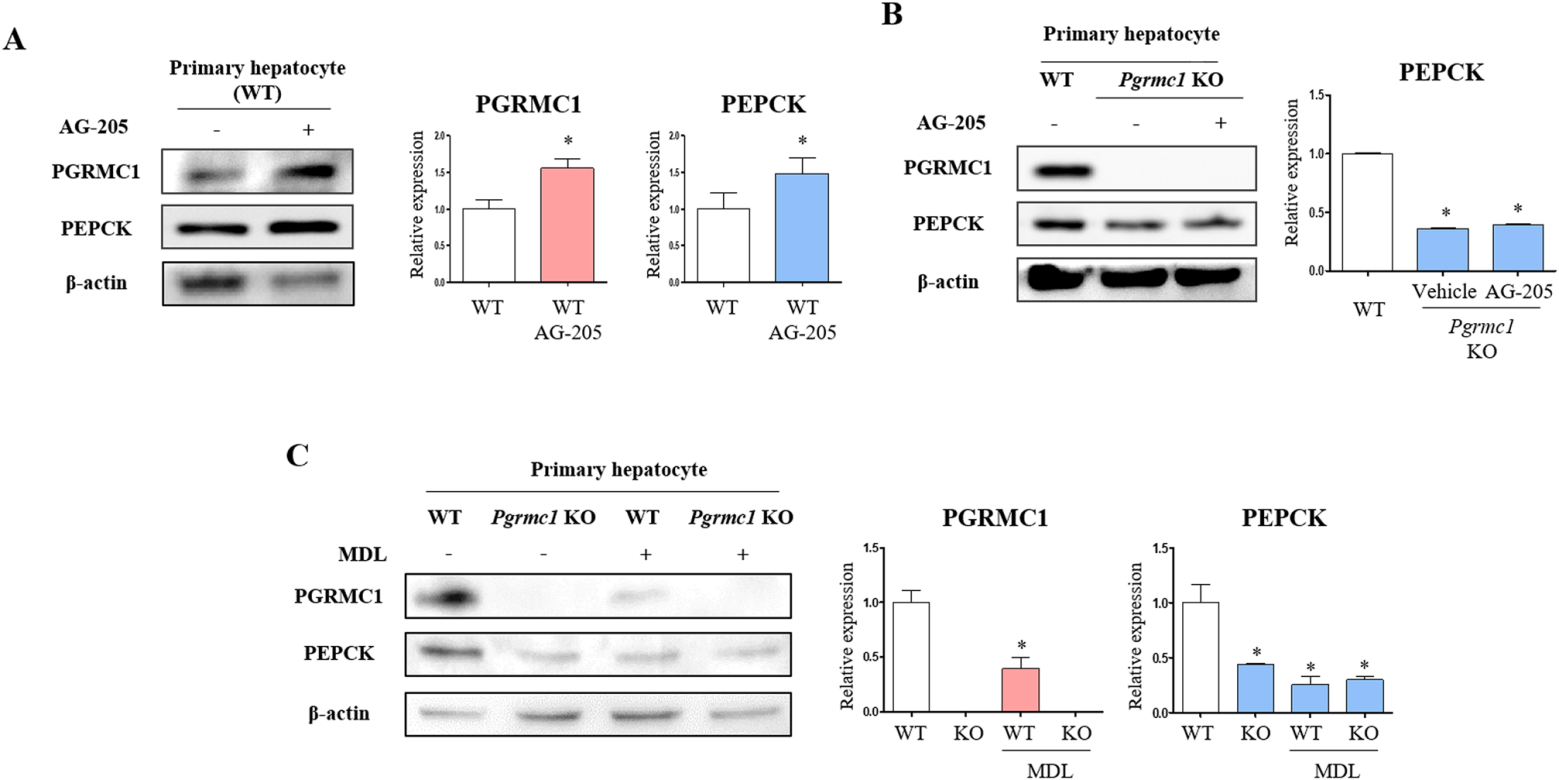
PGRMC1 regulates PEPCK via adenylate cyclase. (**A**) Western blot analysis and quantification of PGRMC1 and PEPCK in WT versus WT + AG-205 hepatocyte. AG-205 (10 µg/ml) was treated in DMEM-low glucose medium for 18 h. β-actin was used as an internal control. Values represent means ± SD. *p < 0.05 versus WT hepatocyte. (**B**) Western blot analysis and quantification of PGRMC1 and PEPCK in WT versus *Pgrmc1* KO versus *Pgrmc1* KO + AG-205 hepatocyte. AG-205 (10 µg/ml) was treated in DMEM-low glucose medium for 18 h. β-actin was used as an internal control. Values represent means ± SD. *p < 0.05 versus WT hepatocyte. (**C**) Western blot analysis and quantification of PGRMC1 and PEPCK in WT, *Pgrmc1* KO, WT + MDL (20 µM), and *Pgrmc1* KO + MDL (20 µM) groups. β-actin was used as an internal control. Values represent means ± SD. *p < 0.05 versus WT hepatocyte. All experiments were repeated at least 3 times.

To gain an insight into the mechanism of regulation of PGRMC1 and PEPCK expression, an adenylate cyclase inhibitor (MDL) was used to determine whether PGRMC1-mediated cAMP accumulation is due to adenylate cyclase activity. As a result, PGRMC1 expression was increased (*p* < 0.05, 1.52-vs. control) in the AG-205 group and remained high (*p* < 0.05, 1.56-fold vs. control) in the AG-205 + MDL group (Fig. 2A). PEPCK expression was also increased (*p* < 0.05, 1.24-fold vs. control) in the AG-205 group, but the increase was suppressed (*p* < 0.05, 72% vs. AG-205 group) in the AG-205 + MDL group (Fig. 2A). While cAMP level was suppressed in the MDL group (*p* < 0.05, 56.5% vs. control), the induction of cAMP was observed in the AG-205 group (*p* < 0.05, 1.24-fold vs. control), and the induction through AG-205 was blocked by MDL treatment (*p* < 0.05, 68.6% vs. AG-205 group), as shown in Fig. 2B. We therefore confirmed the involvement of adenylate cyclase in the PGRMC1-cAMP-PEPCK expression pattern; an illustration of the suggested pathway is provided in Fig. 2C. To confirm PGRMC1-mediated PEPCK induction by AG-205, we additionally monitored the expression of PGRMC1 monomer and PEPCK in primary WT and *Pgrmc1* KO hepatocytes with AG-205. Following induction (*p* < 0.05, 1.56-fold vs. control) of PGRMC1 by AG-205, the PEPCK protein was also increased (*p* < 0.05, 1.48-fold vs. control) in primary hepatocytes (Fig. 3A). Importantly, the expression of PEPCK was decreased (*p* < 0.05, 36.1% vs. WT hepatocytes) in *Pgrmc1* KO primary hepatocytes, and AG-205 was not sufficient to increase PEPCK expression (Fig. 3B). Through these results, we confirmed that AG-205 regulates PEPCK via the PGRMC1 monomer abundance. To confirm the involvement of adenylate cyclase, we treated MDL-12,330A in primary hepatocyte. As expected, the expression of PEPCK was suppressed (*p* < 0.05, 43.6%) in *Pgrmc1* KO hepatocyte compared to WT hepatocytes (Fig. 3C). The expression of PGRMC1 was also inhibited in WT hepatocyte + MDL-12,330A (*p* < 0.05, 39.4% vs. WT control hepatocytes), therefore, PGRMC1 might be related to adenylate cyclase (Fig. 3C). However, after MDL-12,330A treatment, a clear reduction of PEPCK expression was not observed in the *Pgrmc1* KO hepatocyte, which was seen in the WT hepatocyte (Fig. 3C).

### PEPCK regulation by PGRMC1 in hepatocytes

**Figure 4:**
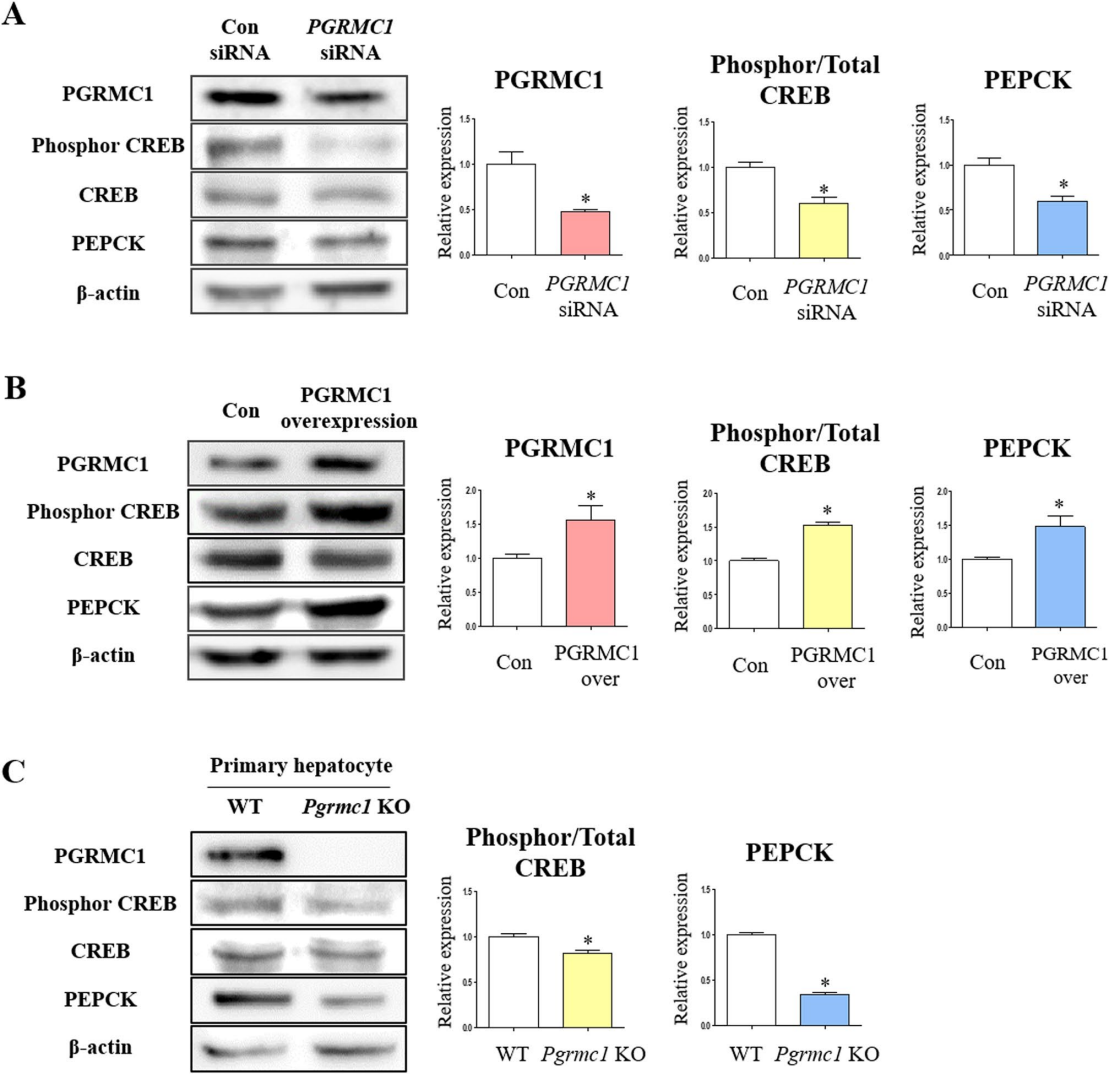
PGRMC1 regulates PEPCK via phosphorylation of CREB. (**A**) Western blot analysis and quantification of PGRMC1, phosphorylated/total CREB, and PEPCK in Control siRNA (Con) versus *PGRMC1* siRNA transfection group. After *PGRMC1* knockdown, cells were incubated in DMEM-low glucose medium (50 mg/dl) for 18 h. β-actin was used as an internal control. Values represent means ± SD. *p < 0.05 versus Con. (**B**) Western blot analysis and quantification of PGRMC1, phosphorylated/total CREB, and PEPCK in Control (Con) versus PGRMC1 overexpression group. After plasmid transfection, cells were incubated in DMEM-low glucose medium for 18 h. β-actin was used as an internal control. Values represent means ± SD. *p < 0.05 versus Con. (**C**) Western blot analysis and quantification of PGRMC1, phosphorylated/total CREB, and PEPCK in WT versus *Pgrmc1* KO hepatocytes. Cells were incubated in DMEM-low glucose medium for 18 h. β-actin was used as an internal control. Values represent means ± SD. *p < 0.05 versus WT hepatocyte. All experiments were repeated at least 3 times.

As PGRMC1 regulates PEPCK according to adenylate cyclase activity, we next focused on CREB phosphorylation which is crucial initiator for PEPCK induction. To evaluate the effect of PGRMC1 on phosphor-CREB and PEPCK expression, we performed *PGRMC1* knockdown in Hep3B cells in low-glucose medium. When the expression of PGRMC1 was decreased (47.6% vs. control) by *PGRMC1* siRNA, the expression of PEPCK was also decreased (*p* < 0.05, 60.2% vs. control) in the *PGRMC1* siRNA group (Fig. 4A). The ratio of phosphorylated/total CREB was concomitantly decreased (*p* < 0.05, 60.6% vs. control) in the *PGRMC1* siRNA group (Fig. 4A). Consistently, the expression of phosphorylated/total CREB and PEPCK and was decreased (*p* < 0.05, 62% and 70.6%, respectively, vs. control) in *PGRMC1* siRNA group even after P4 treatment (Fig. S2). When the expression of PGRMC1 was increased (*p* < 0.05, 1.56-fold vs. control) using a PGRMC1 overexpression vector, the ratio of phosphorylated/total CREB and expression of PEPCK was also increased (*p* < 0.05, 1.53- and 1.48- fold, respectively, vs. control) in the PGRMC1 overexpression group (Fig. 4B). Based on these results, we confirmed that hepatic PGRMC1 can directly control the expression levels of PEPCK protein via phosphorylation of CREB without the influence of hormones or growth factors.

Using primary hepatocytes from WT or *Pgrmc1* KO mice, we confirmed the relationship between PGRMC1 and PEPCK expression in a more physiologically relevant condition. Hepatocytes were incubated in low glucose medium (50 mg/dl) for 24 h. Firstly, the mRNA expression of murine *Pepck* was suppressed (*p* < 0.05, 21.7% vs. WT hepatocytes) in *Pgrmc1* KO primary hepatocytes (Fig. S3). Moreover, the ratio of phosphorylated/total CREB and expression of PEPCK were suppressed (*p* < 0.05, 81.8%, and 34.4%, respectively, vs. WT hepatocytes) in *Pgrmc1* KO hepatocytes (Fig. 4C).

### Progesterone increases PGRMC1, PEPCK, and gluconeogenesis in Hep3B cells

**Figure 5:**
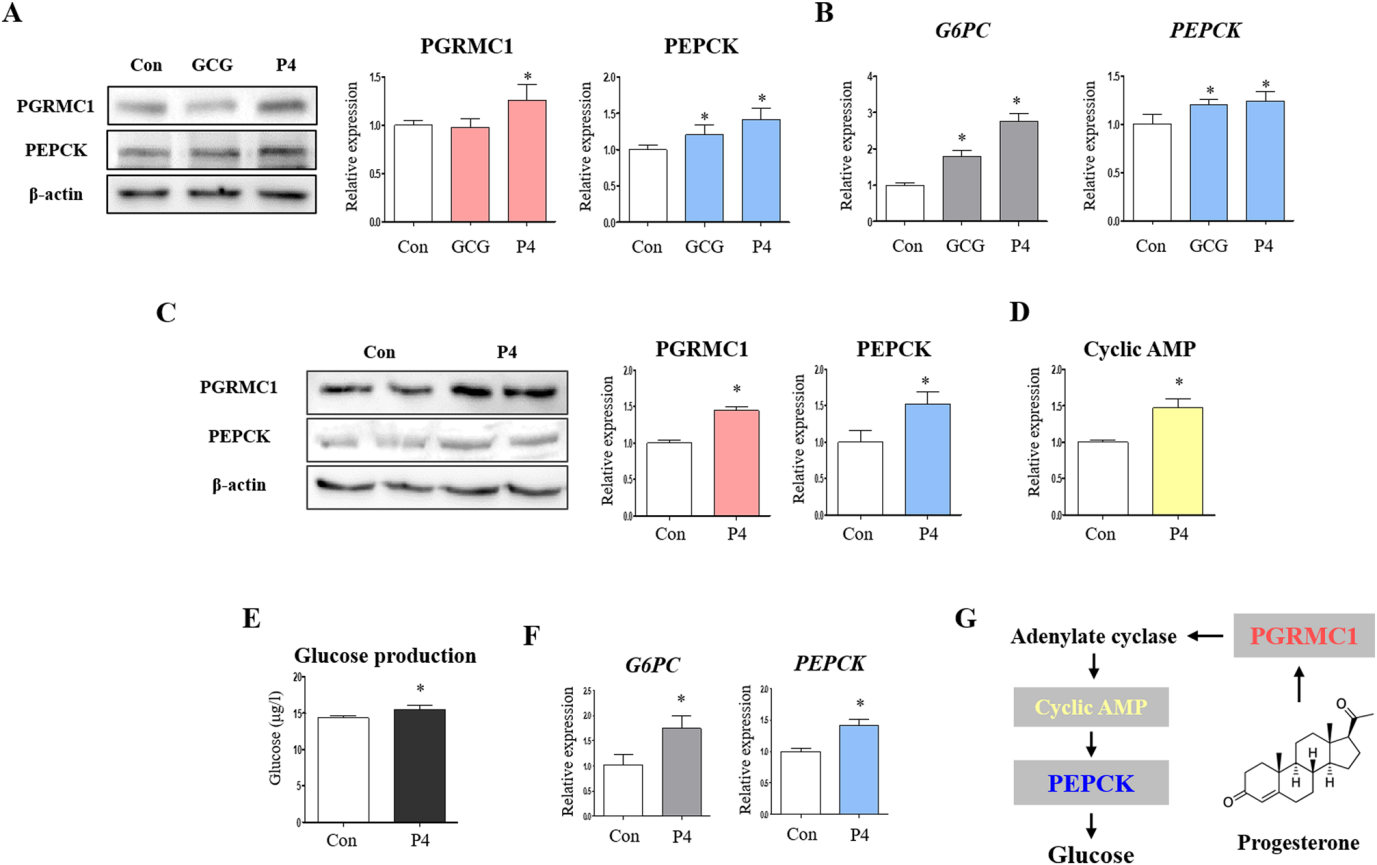
Progesterone increases PGRMC1, phosphorylation of CREB, PEPCK, and gluconeogenesis in Hep3B cell. (**A**) Western blot analysis and quantification of PGRMC1 and PEPCK in Control, GCG (100 µM), and P4 (10 nM) group. β-actin was used as an internal control. Values represent means ± SD. *p < 0.05 versus Control. (**B**) mRNA expression of *G6PC* and *PEPCK* in Control, GCG (100 µM), and P4 (10 nM) group. *RPLP0* was used as an internal control. Values represent means ± SD. *p < 0.05 versus Control. (**C**) Western blot analysis and quantification of PGRMC1 and PEPCK in Control versus P4 group. Cells were harvested when glucose production is measured. β-actin was used as an internal control. Values represent means ± SD. *p < 0.05 versus Control. (**D**) Cyclic AMP (cAMP) levels after 18 h incubation in low glucose medium (50 mg/dl, 2% CD-FBS, 1% Penicillin/Streptomycin). Values represent means ± SD. *p < 0.05 versus Control. (**E**) Glucose production was measured by glucose peroxidase assay. After progesterone pre-treatment (10 nM) for 48 h, cells were incubated with glucose production buffer (sodium pyruvate 2 mM, dexamethasone 1 nM, w/o glucose) for 18 h. Values represent means ± SD. *p < 0.05 versus Control. (**F**) mRNA expression of *G6PC* and *PEPCK* in Control versus P4 group. *RPLP0* was used as an internal control. Values represent means ± SD. *p < 0.05 versus Control. All experiments were repeated at least 3 times. (**G**) Illustration of mechanism between progesterone and gluconeogenesis.

To investigate the relationship between PGRMC1, PEPCK, and steroid hormones, we treated Hep3B cells cultured in low-glucose medium with high dose of 17β-oestradiol (E2, 100 nM) and P4 (100 nM) and had dexamethasone- and AG-205-treated cells as an experimental control. While dexamethasone was not sufficient to induce PGRMC1 and PEPCK expression, the expression of PGRMC1 and PEPCK was increased (*p* < 0.05, 2.86- and 1.72-fold, respectively, vs. control) in the AG-205 group (Fig. S4). Importantly, the expression of PGRMC1 and PEPCK was increased (*p* < 0.05, 3.13- and 1.76-fold, respectively, vs. control) in the P4 group (Fig. S4). Gluconeogenic effect of P4 was assessed with glucagon as a positive control. P4 concentration (10 nM) was set as more physiologic condition. The expression of PGRMC1 was increased (*p* < 0.05, 1.26-fold vs. control) in the P4 group (Fig. 5A). The expression of PEPCK was increased (*p* < 0.05, 1.21- and 1.41, respectively, vs. control) in both GCG and P4 group (Fig. 5A). Transcript level of *G6PC* was increased (*p* < 0.05, 1.79- and 2.76-fold, respectively, vs. control) in the GCG and P4 group (Fig. 5B). Also, mRNA level of *PEPCK* was increased (*p* < 0.05, 1.2- and 1.24-fold, respectively, vs. control) in the GCG and P4 group (Fig. 5B). This result was consistent with that of Hep3B cells in glucose-deprived medium. The expression of PGRMC1 and PEPCK was increased (*p* < 0.05, 1.45- and 1.52-fold, respectively, vs. control in glucose-deprived medium) in the P4 group in glucose-deprived medium (Fig. 5C). Intracellular cAMP was increased (*p* < 0.05, 1.47-fold vs. control) in the P4 group (Fig. 5D). Furthermore, glucose production was increased (*p* < 0.05, 1.08-fold vs. control without glucose) in the P4 group (Fig. 5E). Transcript levels of *G6PC* and *PEPCK* were increased (*p* < 0.05, 1.72- and 1.42-fold, respectively, vs. control) in the P4 group in glucose-deprived medium (Fig. 5F). We observed the induction of PGRMC1-PEPCK-gluconeogenesis by P4 (Fig. 5G).

### Progesterone induces gluconeogenesis in mice in which insulin effects are retarded

**Figure 6:**
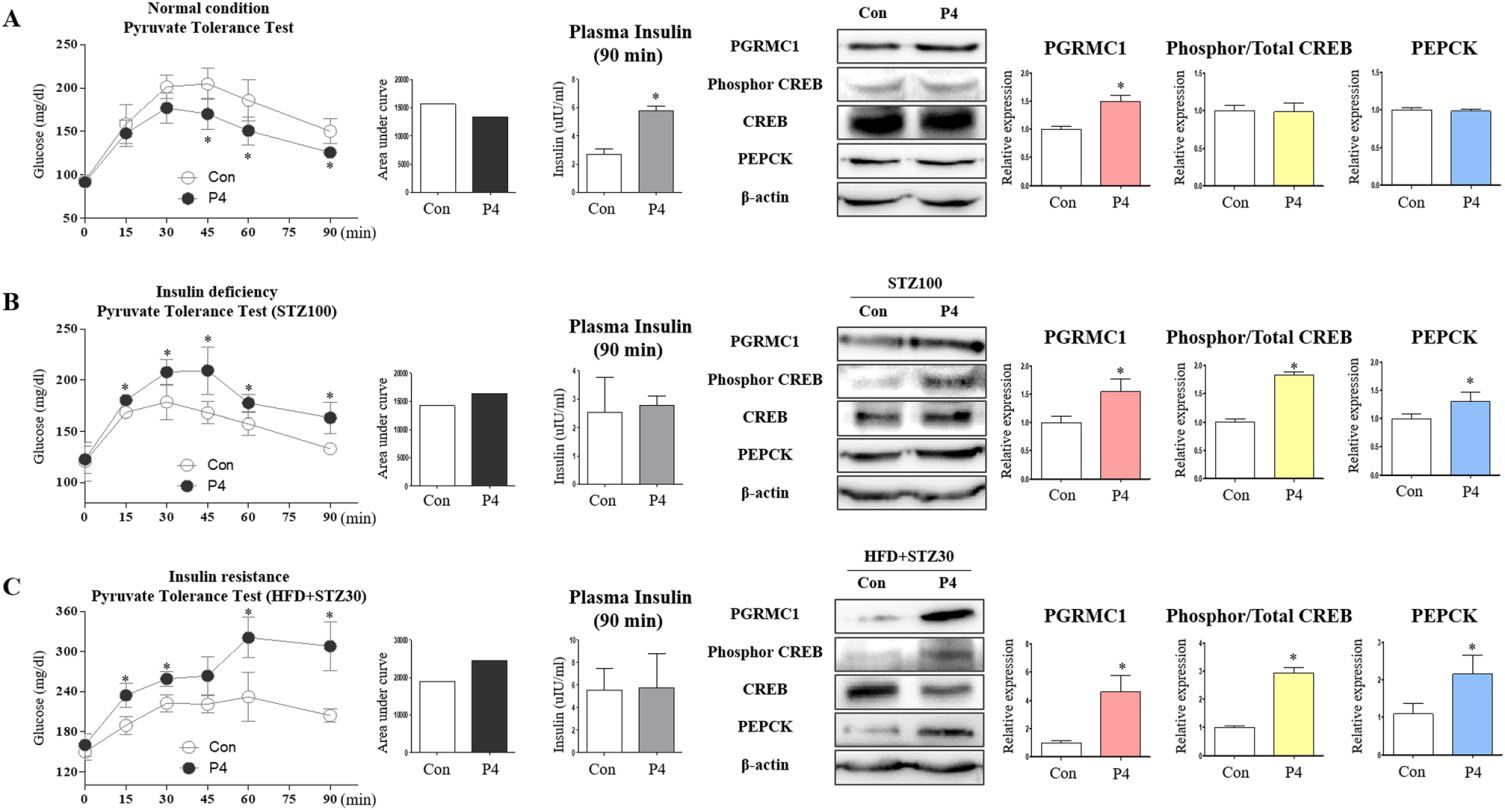
Progesterone induces gluconeogenesis in state which insulin has almost no interference. (**A**) Pyruvate tolerance test was performed after 24 h of fasting. Sodium pyruvate (2 g/kg) was dissolved in PBS and injected into intraperitoneal cavity. AUC was measured. Insulin level was measured after 24 h of fasting and following PTT. Western blot analysis and quantification of PGRMC1, phosphorylated/total CREB, and PEPCK in livers of Control versus P4 group. β-actin was used as an internal control. Values represent means ± SD. *p < 0.05 versus Control. (**B**) Mice were administered high-dose of streptozotocin (100 mg/kg). Pyruvate tolerance test was performed after 24 h of fasting. AUC was measured. Insulin level was measured after 24 h of fasting and following PTT. Western blot analysis and quantification of PGRMC1, phosphorylated/total CREB, and PEPCK in livers of STZ100 versus P4 + STZ100 group. β-actin was used as an internal control. Values represent means ± SD. *p < 0.05 versus Control. (**C**) Mice were administered high-fat diet and low-dose of streptozotocin (30 mg/kg). Pyruvate tolerance test was performed after 24 h of fasting. AUC was measured. Insulin level was measured after 24 h of fasting and following PTT. Western blot analysis and quantification of PGRMC1, phosphorylated/total CREB, and PEPCK in livers of HFD + STZ30 versus P4 + HFD + STZ30 group. β-actin was used as an internal control. Values represent means ± SD. *p < 0.05 versus Control.

We investigated whether P4 increases gluconeogenesis in vivo via PGRMC1 induction. According to previous studies, while P4 is known to induce gluconeogenic genes, it also promotes insulin secretion in the pancreas. We compared the effects of P4 on gluconeogenesis in normal, insulin-deficient, and insulin-resistant states. Under typical physiological conditions, P4 suppressed the levels of blood glucose in a pyruvate tolerance test. Blood glucose levels were decreased (*p* < 0.05, 83.1, 81.1, and 83.6%, respectively, vs. control) at 45, 60, and 90 min in the P4 group after sodium pyruvate injection (Fig. 6A). The area under the curve (AUC) was also decreased in the P4-treated group (Fig. 6A). Blood glucose levels were decreased after 45 min, which implies that P4 promoted insulin action because blood glucose levels in the P4 group were similar to those of the control at 15 min. Consistently, the level of plasma insulin (μIU/min) was increased (*p* < 0.05, 2.16-fold vs. control) in the P4 group (Fig. 6A). The expression of PGRMC1 was increased (*p* < 0.05, 1.5-fold vs. control), but the ratio of phosphorylated/total CREB and PEPCK expression were not changed (Fig. 6A).

Conversely, when we triggered insulin deficiency by high-dose of STZ (100 mg/kg), the levels of blood glucose were increased (1.07-, 1.16-, 1.24-, 1.13-, and 1.23-fold, respectively, vs. control) at 15, 30, 45, 60, and 90 min in the P4 group after pyruvate injection (Fig. 6B). The AUC was also increased in the P4 group (Fig. 6B). Plasma insulin levels were similarly low between the control and P4 groups (Fig. 6B). The expression of PGRMC1, the ratio of phosphorylated/total CREB, and the expression of PEPCK were increased (*p* < 0.05, 1.54-, 1.83-, 1.31-fold, respectively, vs. control) in the P4 group (Fig. 6B).

Similarly, when we triggered insulin resistance by feeding mice a high-fat diet and injecting them with a low dose of STZ (30 mg/kg), the levels of blood glucose were increased (1.24-, 1.16-, 1.38-, and 1.51-fold, respectively, vs. control) at 15, 30, 60, and 90 min in the P4 group after pyruvate injection (Fig. 6C). The AUC was also increased in the P4 group (Fig. 6C). Plasma insulin levels were similar between the control and P4 groups (Fig. 6C). The expression of PGRMC1, the ratio of phosphorylated/total CREB, and the expression of PEPCK were increased (*p* < 0.05, 4.61-, 2.93-, and 1.97-fold, respectively, vs. control) in the P4 group (Fig. 6C). Collectively, these results are consistent with in vitro data in the present study, showing the induction of hepatic gluconeogenesis by P4 when insulin action was limited.

## Discussion

Previous research has reported that a high dose of P4 disturbs glucose homeostasis linked with diabetes mellitus^15^. Moreover, P4 receptor-knockout mice were less prone to hyperglycaemia^16^. While P4 seems to be the reason for insulin resistance that triggers diabetes mellitus during gestation^15^, the role of P4 in gluconeogenesis remains unclear. In our study, we identified the relationship between PGRMC1 and PEPCK and further examined the effect of P4 on gluconeogenesis via PEPCK induction. AG-205 increased PEPCK expression via PGRMC1 and adenylate cyclase, and the PGRMC1-mediated PEPCK expression involved with phosphorylation of CREB. Consistent with a previous study reporting that P4 interacts with PGRMC1^17^, P4 increased PGRMC1 and PEPCK expression, cAMP level, and glucose production. These observations are important because the role of PGRMC1 could be highlighted with direct evidence by modulating PGRMC1 in vitro. Following PTT analysis in a mouse model, P4 increased gluconeogenesis via induction of PGRMC1 and PEPCK when insulin action is limited. Our study suggests that PGRMC1 may be an emerging target for controlling gluconeogenesis and diabetes in an insulin-resistant state.

Using siRNA or overexpression plasmids regulating PGRMC1 levels, we confirmed that hepatic PGRMC1 regulates PEPCK, a key enzyme in gluconeogenesis. Moreover, the PGRMC1 ligand AG-205 accumulates PGRMC1 monomer, and this induction of PGRMC1 monomer enhanced PEPCK expression through cAMP accumulation. Although glucagon and epinephrine are also known to induce intracellular cAMP production and PKA activation^18^, intracellular cAMP accumulation occurred upon an increase in PGRMC1 levels without glucagon or epinephrine in our study. In addition, MDL-12,330A completely inhibited expression of PEPCK in both WT and *Pgrmc1* KO hepatocytes (Fig. 3C), suggesting PGRMC1 regulates PEPCK through cAMP and adenylate cyclase dependent manner. After the increase in cAMP, activated PKA then enters the nucleus, phosphorylates the cAMP regulatory element-binding protein (CREB), and induces *Pepck* transcription^19^. Knockdown of hepatic CREB substantially reduced blood glucose production in a ZDF T2DM rat model^20^. PGRMC1 regulates PEPCK expression via CREB phosphorylation, considering the results of PGRMC1 knockdown and overexpression in Hep3B cells. It was also confirmed that the PGRMC1 regulates the ratio of phosphor/total CREB and expression of PEPCK in primary hepatocytes. Therefore, increased cAMP production through the induced increase in PGRMC1 levels will lead to an increased rate of gluconeogenesis.

AG-205 is known as a PGRMC1 inhibitor based on many previous studies but has also been shown to increase PGRMC1 monomer levels^21^. In addition, a previous study showed that AG-205 treatment decreased dimeric and oligomeric forms of PGRMC1 but increased the monomeric form of PGRMC1^22^. Importantly, the PGRMC1 monomer could also be functional, in addition to the multimeric forms of PGRMC1^23^. In the present study, we observed induction of PGRMC1 monomers (25 kDa) after AG-205 treatment, similar to previous reports. We confirmed that increased levels of PEPCK after AG-205 treatment were consistent with results representing PGRMC1 knockdown and overexpression, respectively. Furthermore, it was clear that AG-205 induced PEPCK through induction of PGRMC1 monomer because PEPCK levels in PGRMC1 KO hepatocytes did not increase upon AG-205 treatment.

A derivative of P4, 17α-hydroxyprogesterone, can lower the occurrence rate of preterm delivery^24^, which is prevalent in moderately overweight and obese pregnant women^25^. However, in a clinical study, women treated with 17α-hydroxyprogesterone caproate during pregnancy were predisposed to gestational diabetes compared to the control group^26^. While P4 contributes to insulin secretion^8,9^, it can also result in insulin resistance and the concurrent induction of gluconeogenic genes^7^. To exclude the effect of P4-mediated insulin, we used in vitro conditions to investigate the role of P4 in hepatocytes. In vitro P4 increased PGRMC1, PEPCK, cAMP, and glucose production. However, P4 seems to suppress gluconeogenic activity in vivo as P4 promotes insulin secretion in normal mice. Interestingly, P4 also induces phosphorylation of CREB, PEPCK, and gluconeogenesis in mice under conditions of insulin-deficient and insulin-resistant conditions. Accordingly, P4 induces hepatic gluconeogenesis and maintains high glucose levels in a state that insulin has almost no effect due to insulin resistance. This is important as normal pregnancy usually includes insulin resistance^27^, and P4 might induce gestational diabetes by increasing gluconeogenesis when the effects of insulin are not considered.

Type II diabetes is characterized by hyperglycaemia during a fasting state and by a prolonged rise in blood glucose levels after the fed state. This type of diabetes is associated with insulin resistance and promotes gluconeogenesis despite high insulin levels^28^. Furthermore, increased alpha-cell function and consequent hyperglucagonemia have been recognized as contributors to hyperglycaemia in type II diabetic patients because of gluconeogenesis activation^29^. In an early study, gestational P4 had relative diabetogenic properties, decreased glucose uptake, and increased hepatic glucose production^15^. It is very interesting that treatment of pregnant women with P4 for the prevention of recurrent preterm birth increased the risk of gestational diabetes mellitus^26^. In this study, we demonstrated that P4-mediated PGRMC1 induction increases gluconeogenesis under insulin-resistant conditions such as type II diabetes. In conclusion, our study suggests that the regulation of gluconeogenesis by PGRMC1 modulation holds great potential as a new therapeutic approach to manage and treat type II diabetes.

## Methods

### Chemicals

MDL-12,330A hydrochloride was purchased from Tocris Bioscience. Progesterone was purchased from Tokyo Chemical Industry. Streptozotocin was purchased from Santa Cruz Biotechnology. Sodium pyruvate, glucagon, and dexamethasone were purchased from Sigma-Aldrich.

### Animals

C57BL/6J WT and *Pgrmc1* KO male mice were housed in a pathogen-free facility at Chungnam National University under a standard 12 h light:12 h dark cycle and fed standard chow with water provided ad libitum. All mouse experiments were approved and performed in accordance with the Chungnam Facility Animal Care Committee (CNU-00606).

For the pyruvate tolerance test (PTT), mice were injected with P4 (1 mg/kg) for 3 days and fasted for 24 h as of the last injection. Mice were injected with sodium pyruvate (2 g/kg), and blood glucose was monitored by tail snipping. For insulin-deficiency, mice were pre-treated with streptozotocin (STZ) (100 mg/kg) before 1 week and were assessed by PTT. For the insulin-resistant state, mice were fed a high-fat diet for 8 weeks. On the first day of week 6 of the high-fat diet, mice were injected with a low dose of STZ (30 mg/kg). After 8 weeks of high-fat diet, mice were assessed by PTT. Streptozotocin was dissolved in citrate buffer (pH 4.4) and injected into the intraperitoneal cavity (IP). Progesterone was injected into the subcutaneous inguinal area. A high-fat diet (Research Diets, New Brunswick, NJ) was composed of carbohydrate (20% kcal), protein (20% kcal), and fat (60% kcal).

### Blood glucose level

Blood glucose levels were measured with the Accu-Chek Active kit (Roche) without any restraint to mice.

### Cyclic AMP measurement

After cell lysis, cell extracts were processed according to the manufacturer’s protocol (Promega).

### Glucose production assay

Hep3B cells were maintained in glucose-free medium containing 2 mM sodium pyruvate and 1 nM dexamethasone. The medium was prepared according to the manufacturer’s protocol (Sigma-Aldrich).

### RNA isolation, Reverse transcription, and qRT-PCR

RNA isolated by TRIzol Reagent (Thermo Fisher Scientific), chloroform (Sigma-Aldrich), and isopropanol (Merck Millipore) was dissolved in DEPC (Amresco)-treated water. cDNA acquired by Reverse Transcriptase Kit (SG-cDNAS100, Smartgene, United Kingdom), specific primers (shown below), Excel Taq Q-PCR Master Mix (SG-SYBR-500, Smartgene) and Stratagene Mx3000P (Agilent Technologies) were used to perform real-time PCR.

Human primers used were: *PGRMC1* forward (AAA GGC CGC AAA TTC TAC GG), *PGRMC1* reverse (CCC AGT CAC TCA GAG TCT CCT), *PEPCK* forward (GCC ATC ATG CCG TAG CAT C), *PEPCK* reverse (AGC CTC AGT TCC ATC ACA GAT), *G6PC* forward (GTG TCC GTG ATC GCA GAC C), *G6PC* reverse (GAC GAG GTT GAG CCA GTC TC).

Mouse primers used were: *Pgrmc1* forward (GGC AAG GTG TTC GAC GTG A), *Pgrmc1* reverse (GTC CAG GCA AAA TGT GGC AA) *Pepck* forward (CTG CAT AAC GGT CTG GAC TTC), *Pepck* reverse (CAG CAA CTG CCC GTA CTC C).

### Western blotting

Liver and Hep3B cells were lysed and sonicated for protein extraction. Protein samples were subjected to 8–12% SDS-PAGE after 5 min of boiling at 100 °C. Gels were blotted by wet transfer with Bio-Rad Power Pac at 350 V for 1 to 2 h. PVDF membranes were blocked for 1 h in bovine serum albumin (BSA) and incubated with primary antibodies overnight at 4 °C. Membranes were then incubated with secondary antibodies diluted with 1:10,000 in BSA overnight at 4 °C. The results were detected with enhanced chemiluminescence (ECL) solution (Cyanagen) and ChemiDoc (Fusion Solo, Vilber Lourmat). Primary polyclonal antibodies used were: rabbit anti-β-actin (sc-130656, Santa Cruz Biotechnology, USA) and rabbit anti-PEPCK (10004943, Cayman Chemical, USA). Rabbit monoclonal antibody to PGRMC1 (#13856) was purchased from Cell Signaling Technology, Inc. Rabbit monoclonal antibody to phosphor CREB (ab32096, Abcam), CREB (ab32515, Abcam) were used. The secondary antibody used was mouse anti-rabbit IgG (211-032-171 anti-rabbit, Jackson laboratory).

### Cell culture

All cell culture reagents were purchased from Welgene (Gyeongsan, Korea). Hep3B cells were obtained from Korean Cell Line Bank (KCLB, 88064). Hep3B cells and liver primary cells were maintained at 37 °C in a 5% CO_2_ atmosphere in Dulbecco’s Modified Eagle Medium (DMEM) (Welgene) supplemented with 5% (vol/vol) foetal bovine serum, penicillin (100 U/mol) and streptomycin (100 μg/ml). Cells were washed once with Dulbecco’s Phosphate-Buffered Saline (DPBS) (Welgene) and incubated with low glucose medium (50 mg/dl, w/o FBS; Welgene) for 18 h. P4 was treated with CD-FBS for steroid hormone delivery. All cell experiments were repeated at least three times.

For PGRMC1 overexpression, Hep3B cells were transfected with 2.5 µg of human PGRMC1 expression plasmid and Lipofectamine 2000 (Invitrogen) in Opti-MEM (Gibco) medium according to the manufacturer’s protocol. For PGRMC1 knockdown, Hep3B cells were transfected with control siRNA or *PGRMC1* siRNA #1 (5′-CAGUACAGUCGCUAGUCAA-3′) and #2 (5′-CAGUUCACUUUCAAGUAUCA-U-3′) purchased from Bioneer (Daejeon, Korea).

### Primary hepatocyte isolation

Primary cell culture was performed as described previously^30^. The hepatocytes of mice were isolated by collagenase digestion and the Percoll Gradient method. In detail, mice were anesthetized and perfused with Ca^2+^ and Mg^2+^ free-Hanks’ Balanced Salt Solution (HBSS) containing EDTA (1 mM), and then digested with a collagenase solution containing liberase (0540119001, Sigma). Livers were removed and rinsed twice with HBSS, digested with a collagenase solution containing liberase (0540119001, Sigma-Aldrich) and then gently teased with forceps until they were in solution.

The cell suspensions were filtered through a sterile 40 μm nylon cell strainer (93040, SPL) to remove undigested tissue and connective tissue. The cells were centrifuged for 5 min at 1000 rpm and resuspended in medium. The resuspended cells were centrifuged using 40% Percoll for 15 min at 2000 rpm with the brake option off. After centrifugation, the healthy hepatocytes were washed twice with DMEM supplemented with 5% FBS, and then seeded into well tissue culture plates.

### Statistical analysis

Data are reported as the mean ± standard deviation (SD). Differences between means were obtained by Student’s t-test and one-way ANOVA followed by a Dunnett post analysis was performed using Graph Pad Software (GraphPad Inc., San Diego, CA).

## Supplementary information

Supplementary Information.

## References

[1] Choi SI, Lee HA, Han JS (2016). Gynura procumbens extract improves insulin sensitivity and suppresses hepatic gluconeogenesis in C57BL/KsJ-db/db mice. Nutr. Res. Pract..

[2] Edgerton DS (2006). Insulin’s direct effects on the liver dominate the control of hepatic glucose production. J. Clin. Invest..

[3] Exton JH, Park CR (1967). Control of gluconeogenesis in liver. I. General features of gluconeogenesis in the perfused livers of rats. J. Biol. Chem..

[4] Rodgers RL (2012). Glucagon and cyclic AMP: time to turn the page?. Curr. Diabetes Rev..

[5] Quinn PG, Yeagley D (2005). Insulin regulation of PEPCK gene expression: a model for rapid and reversible modulation. Curr. Drug Targets Immune Endocr. Metabol. Disord..

[6] Spencer TE, Bazer FW (2002). Biology of progesterone action during pregnancy recognition and maintenance of pregnancy. Front. Biosci..

[7] Masuyama H, Hiramatsu Y (2011). Potential role of estradiol and progesterone in insulin resistance through constitutive androstane receptor. J. Mol. Endocrinol..

[8] Costrini NV, Kalkhoff RK (1971). Relative effects of pregnancy, estradiol, and progesterone on plasma insulin and pancreatic islet insulin secretion. J. Clin. Invest..

[9] Ashby JP, Shirling D, Baird JD (1978). Effect of progesterone on insulin secretion in the rat. J. Endocrinol..

[10] Oda S, Nakajima M, Toyoda Y, Fukami T, Yokoi T (2011). Progesterone receptor membrane component 1 modulates human cytochrome p450 activities in an isoform-dependent manner. Drug Metab. Dispos..

[11] Rohe HJ, Ahmed IS, Twist KE, Craven RJ (2009). PGRMC1 (progesterone receptor membrane component 1): a targetable protein with multiple functions in steroid signaling, P450 activation and drug binding. Pharmacol. Ther..

[12] Lee SR (2018). Loss of progesterone receptor membrane component 1 promotes hepatic steatosis via the induced de novo lipogenesis. Sci. Rep..

[13] Meloni AR, DeYoung MB, Lowe C, Parkes DG (2013). GLP-1 receptor activated insulin secretion from pancreatic beta-cells: mechanism and glucose dependence. Diabetes Obes. Metab..

[14] Zhang M (2014). Progesterone receptor membrane component 1 is a functional part of the glucagon-like peptide-1 (GLP-1) receptor complex in pancreatic beta cells. Mol. Cell Proteom..

[15] Branisteanu DD, Mathieu C (2003). Progesterone in gestational diabetes mellitus: guilty or not guilty?. Trends Endocrinol. Metab..

[16] Picard F (2002). Progesterone receptor knockout mice have an improved glucose homeostasis secondary to beta -cell proliferation. Proc. Natl. Acad. Sci. U. S. A..

[17] Peluso JJ (2013). Progesterone receptor membrane component 1 and its role in ovarian follicle growth. Front. Neurosci..

[18] Moncany ML, Plas C (1980). Interaction of glucagon and epinephrine in the regulation of adenosine 3',5'-monophosphate-dependent glycogenolysis in the cultured fetal hepatocyte. Endocrinology.

[19] Herzig S (2001). CREB regulates hepatic gluconeogenesis through the coactivator PGC-1. Nature.

[20] Erion DM (2009). Prevention of hepatic steatosis and hepatic insulin resistance by knockdown of cAMP response element-binding protein. Cell Metab..

[21] Karicheti V, Langdale CL, Ukai M, Thor KB (2010). Characterization of a spinal, urine storage reflex, inhibitory center and its regulation by 5-HT1A receptors in female cats. Am. J. Physiol. Regul. Integr. Comp. Physiol..

[22] Will EA, Liu X, Peluso JJ (2017). AG 205, a progesterone receptor membrane component 1 antagonist, ablates progesterone’s ability to block oxidative stress-induced apoptosis of human granulosa/luteal cellsdagger. Biol. Reprod..

[23] Peluso JJ, Liu X, Gawkowska A, Lodde V, Wu CA (2010). Progesterone inhibits apoptosis in part by PGRMC1-regulated gene expression. Mol. Cell Endocrinol..

[24] How HY, Sibai BM (2009). Progesterone for the prevention of preterm birth: indications, when to initiate, efficacy and safety. Ther. Clin. Risk Manag..

[25] Cnattingius S (2013). Maternal obesity and risk of preterm delivery. JAMA.

[26] Rebarber A (2007). Increased incidence of gestational diabetes in women receiving prophylactic 17alpha-hydroxyprogesterone caproate for prevention of recurrent preterm delivery. Diabetes Care.

[27] Sonagra AD, Biradar SM, Dattatreya K, Murthy DSJ (2014). Normal pregnancy—a state of insulin resistance. J. Clin. Diagn. Res..

[28] Basu R, Chandramouli V, Dicke B, Landau B, Rizza R (2005). Obesity and type 2 diabetes impair insulin-induced suppression of glycogenolysis as well as gluconeogenesis. Diabetes.

[29] Menge BA (2011). Loss of inverse relationship between pulsatile insulin and glucagon secretion in patients with type 2 diabetes. Diabetes.

[30] Hong EJ, Levasseur MP, Dufour CR, Perry MC, Giguere V (2013). Loss of estrogen-related receptor alpha promotes hepatocarcinogenesis development via metabolic and inflammatory disturbances. Proc. Natl. Acad. Sci. U. S. A..

