# Supplementary material for: Progesterone increases blood glucose via hepatic progesterone receptor membrane component 1 under limited or impaired action of insulin
Source: Sci Rep. 2020 Oct 1;10:16316. doi: 10.1038/s41598-020-73330-7 (PMC7529793; doi:10.1038/s41598-020-73330-7)

Supplementary material

Progesterone increases blood glucose via hepatic progesterone receptor membrane component 1 under limited or impaired action of insulin

Sang R. Lee[1], Woo-Young Choi[1], Jun H. Heo[1], Jiyoung Huh[1], Globinna Kim[2], Kyu-Pil Lee[1], Hyo-Jung Kwun[1], Hyun-Jin Shin[1], In-Jeoung Baek[2], and Eui-Ju Hong[1*]

[1]College of Veterinary Medicine, Chungnam National University, Daejeon 34134, Republic of Korea; [2]Department of Convergence Medicine, University of Ulsan College of Medicine, Asan Medical Center, Seoul 05505, Republic of Korea

[*]Address correspondence to: Eui-ju Hong, College of Veterinary Medicine, 99Daehak-ro, Suite 401Veterinary medicine Bldg., Yuseong, Daejeon 34134, Korea. Phone: +82-42-821-6781;Fax: +82-42-821-8903 Email: ejhong@cnu.ac.kr

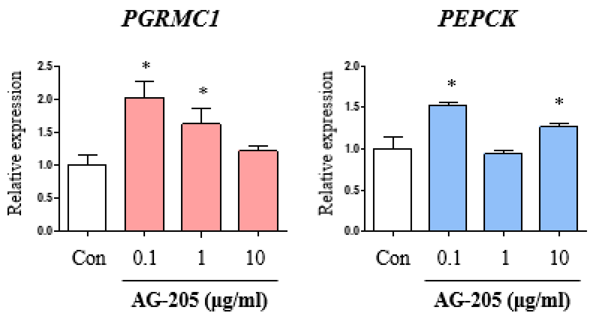

Fig S1. mRNA expression of *PGRMC1* and *PEPCK* in Hep3B cells AG-205 (0.1, 1, 10 μg/ml) was treated in low glucose medium (50mg/dl) for 18 h in Hep3B cells. *RPLP0* was used as an internal control. Values represent means ± SD. *, $p<0.05$ vs. Control. All experiments were repeated at least 3 times.

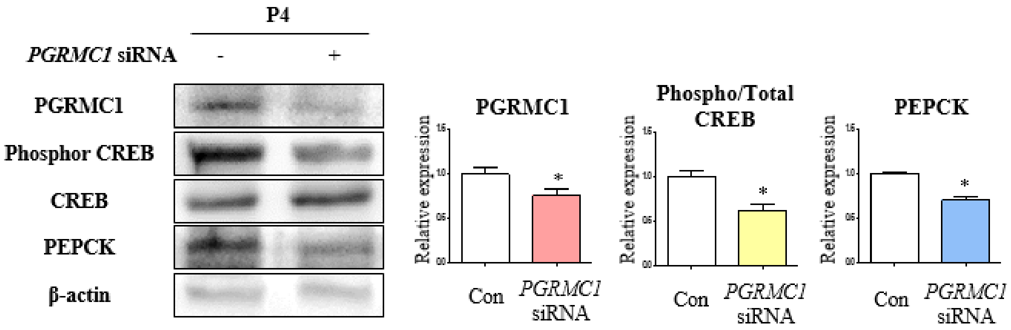

Fig S2. Western blot analysis and quantification of PGRMC1, phosphor/total CREB, and PEPCK in control and *PGRMC1* siRNA groups. Cells were starved in low glucose medium (50 mg/dl). P4 (10 nM) was treated for 18 h after siRNA transfection. β-actin was used as an internal control. Values represent means ± SD. *, $p<0.05$ vs. Control. All experiments were repeated at least 3 times.

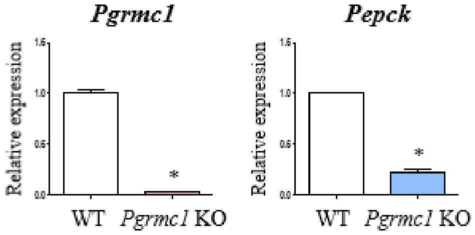

Fig S3. mRNA expression of *Pgrmc1* and *Pepck* in WT and *Pgrmc1* KO hepatocyte. Cells were incubated in low glucose medium (50mg/dl) for 18 h in Hep3B cells. *Rplp0* was used as an internal control. Values represent means ± SD. *, p<0.05 vs. WT hepatocyte. All experiments were repeated at least 3 times.

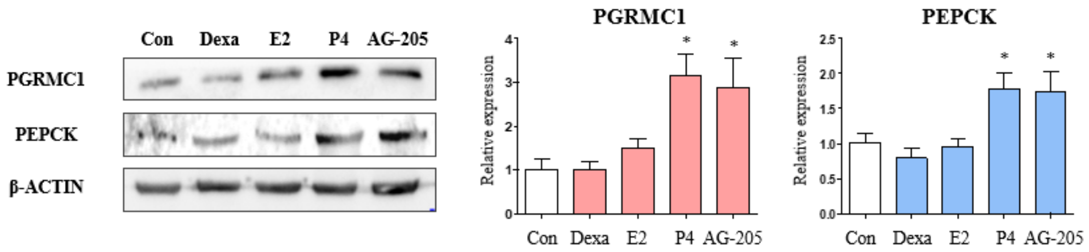

Fig S4. Western blot analysis and quantification of PGRMC1 and PEPCK in Control vs. Dexamethasone, E2, P4, AG-205 groups. Cells were starved in DMEM medium (w/o glucose, w/o FBS). Dexamethasone (100 nM), E2 (100 nM), P4 (100 nM) and AG-205 (10 µg/ml) were treated in DMEM medium (w/o glucose, 2%CD-FBS, 1% Penicillin/Streptomycin, 1nM dexamethasone) for 24 h. β-actin was used as an internal control. Values represent means ± SD. *, $p<0.05$ vs. Control. All experiments were repeated at least 3 times.

Fig.1A

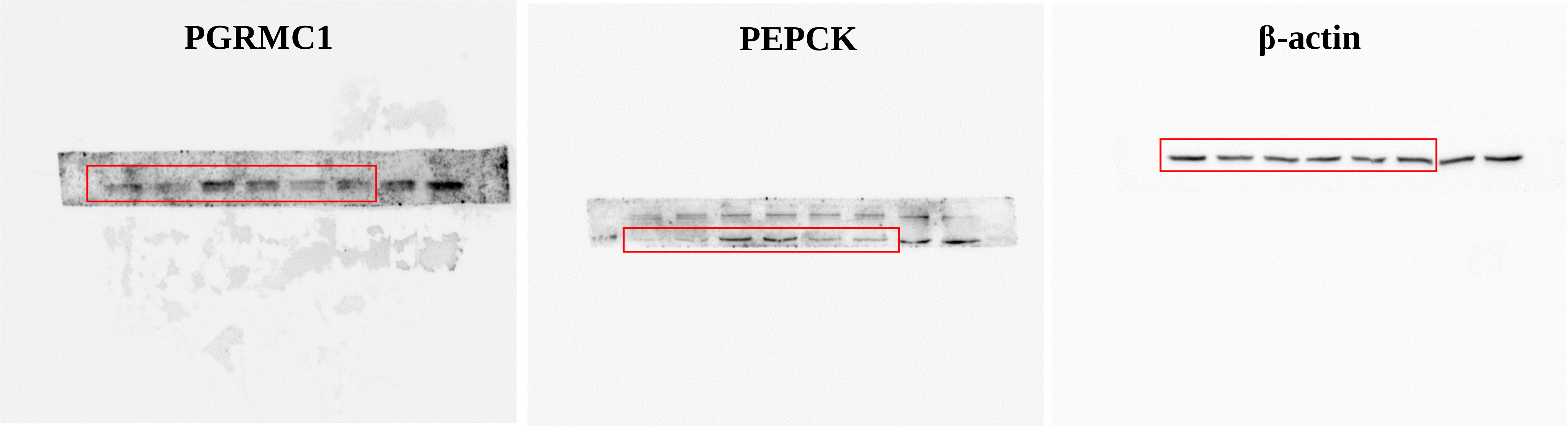

Fig.1B

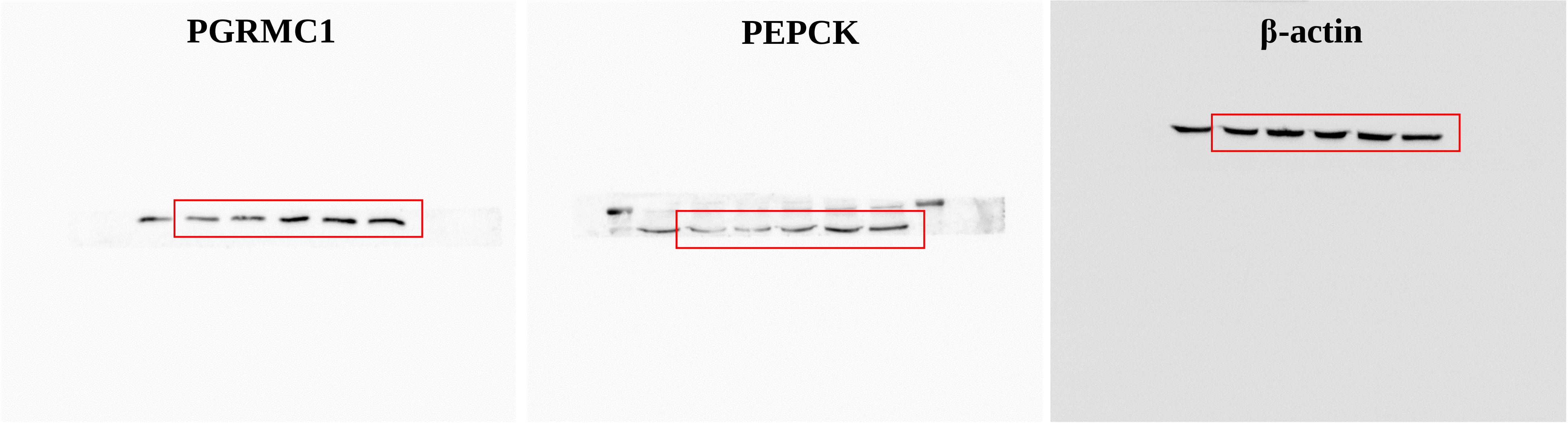

Fig.1D

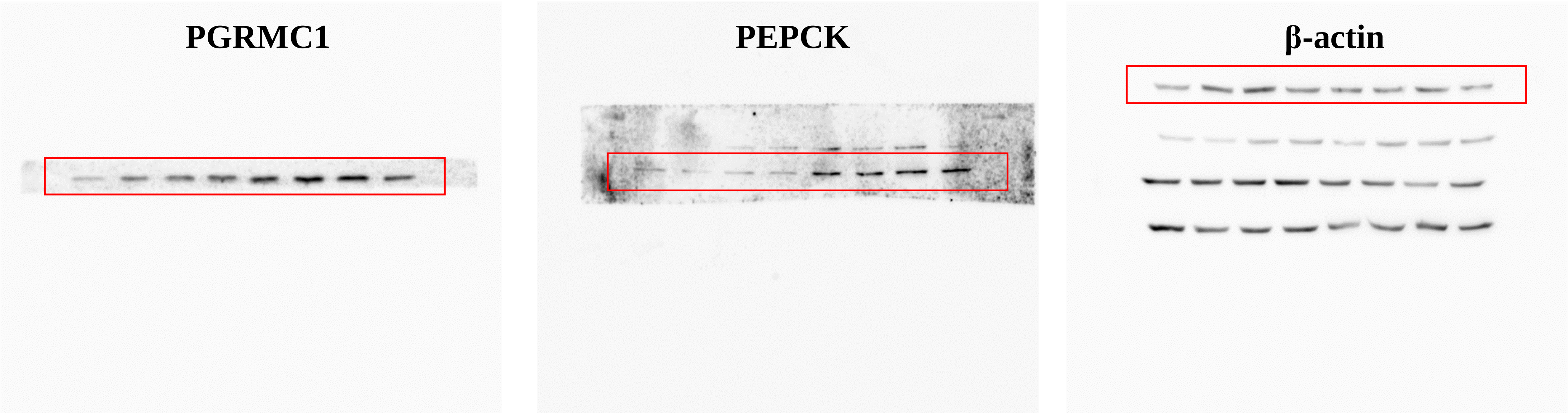

Fig.2A

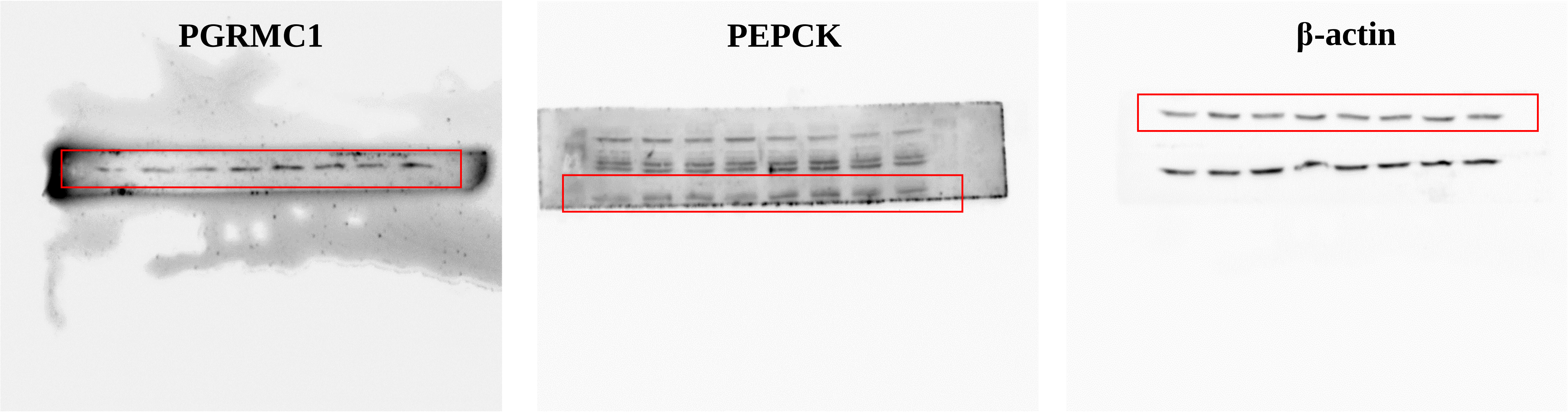

Fig.3A

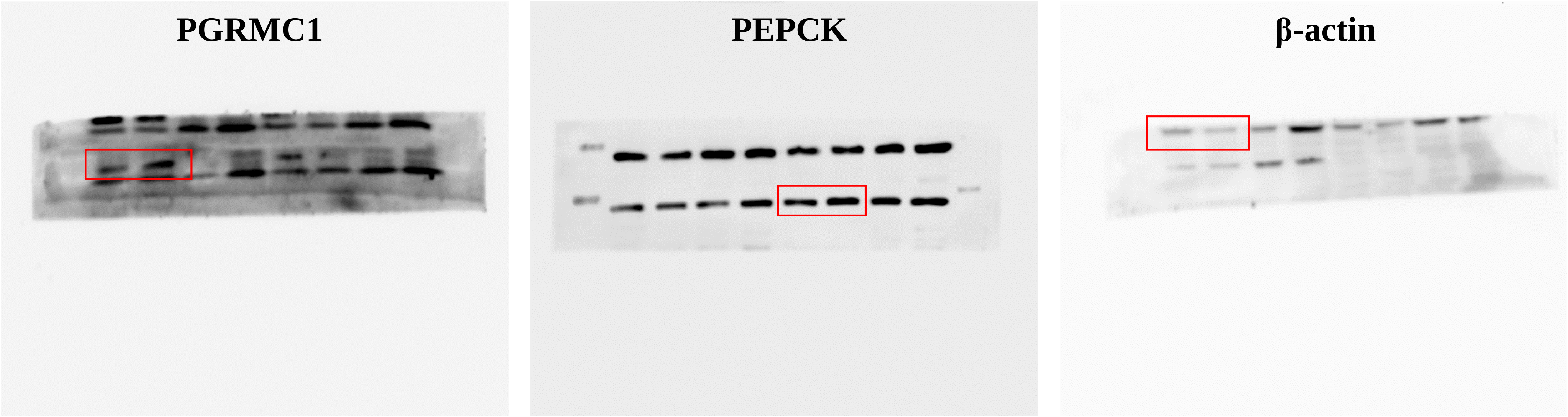

Fig.3B

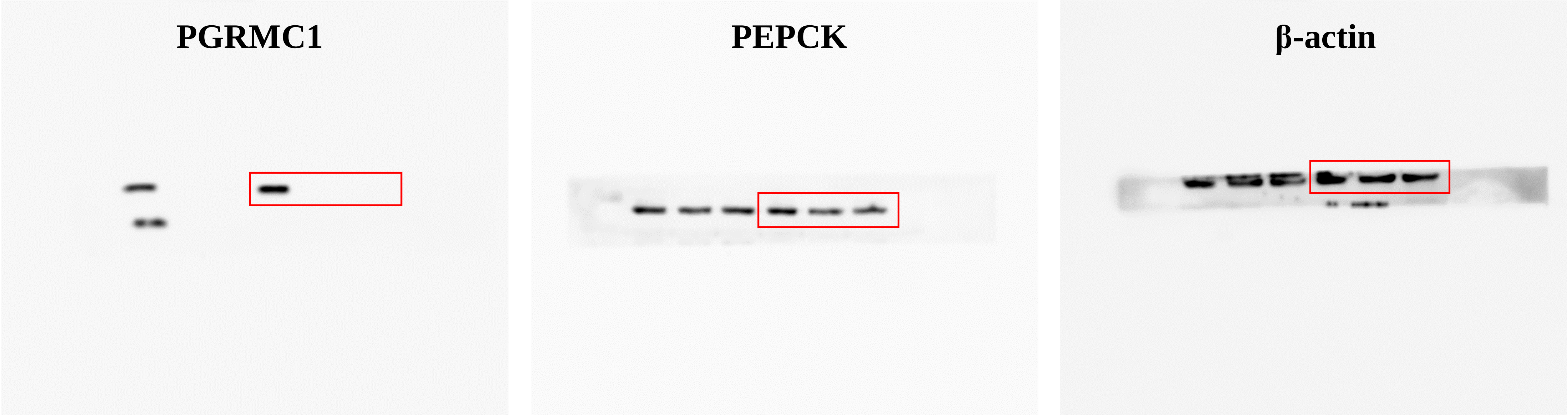

Fig.3C

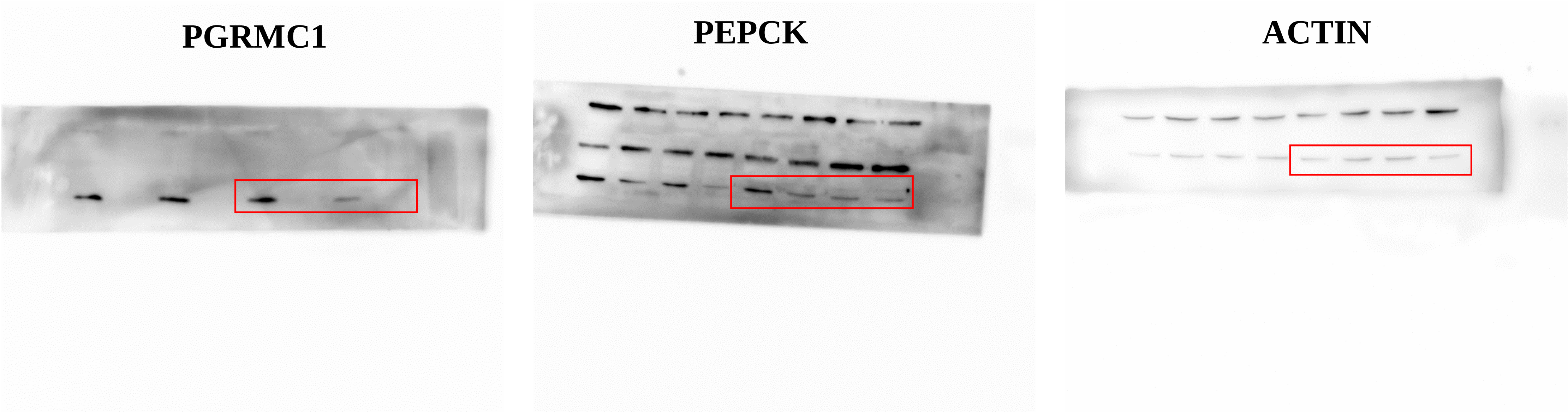

Fig.4A

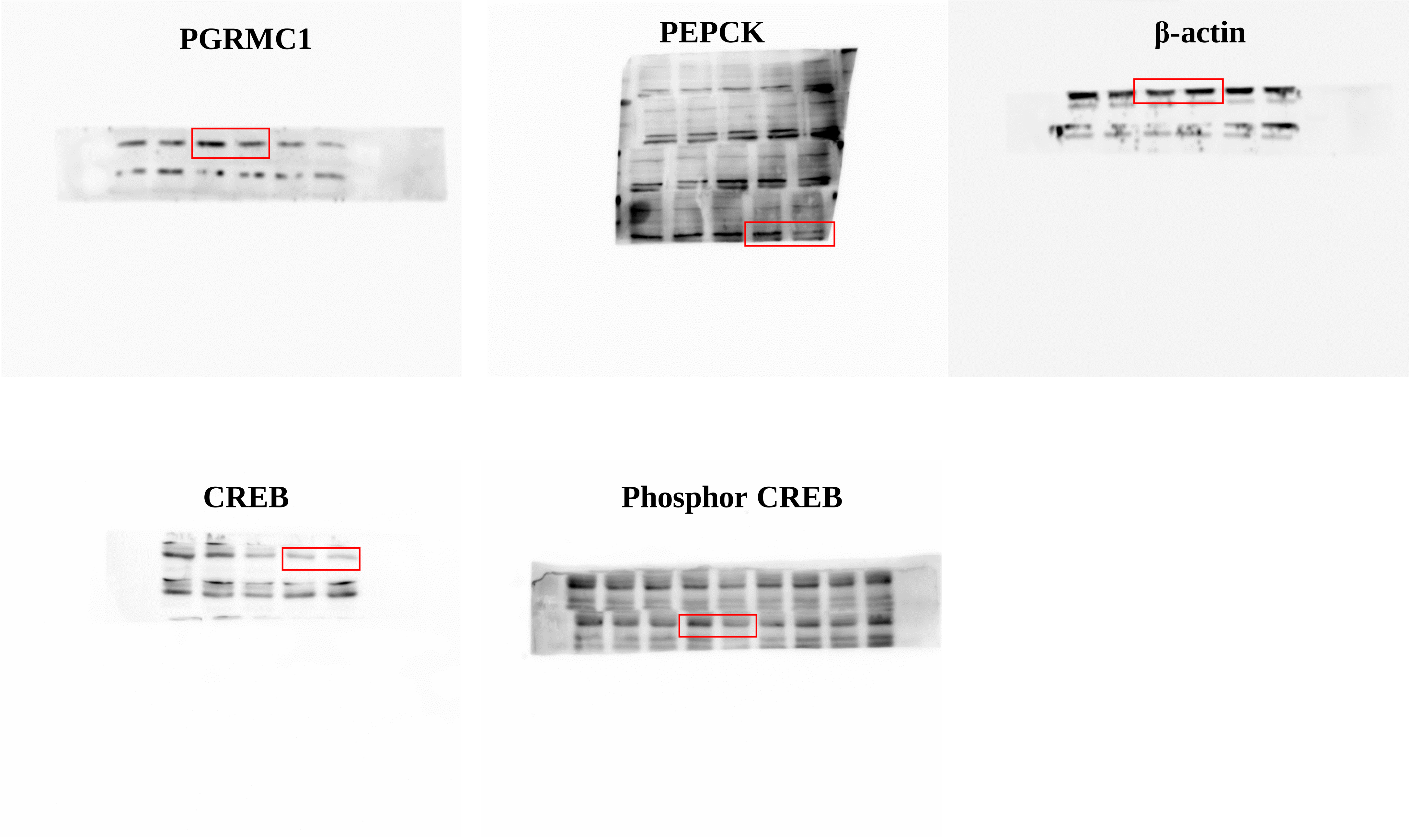

Fig.4B

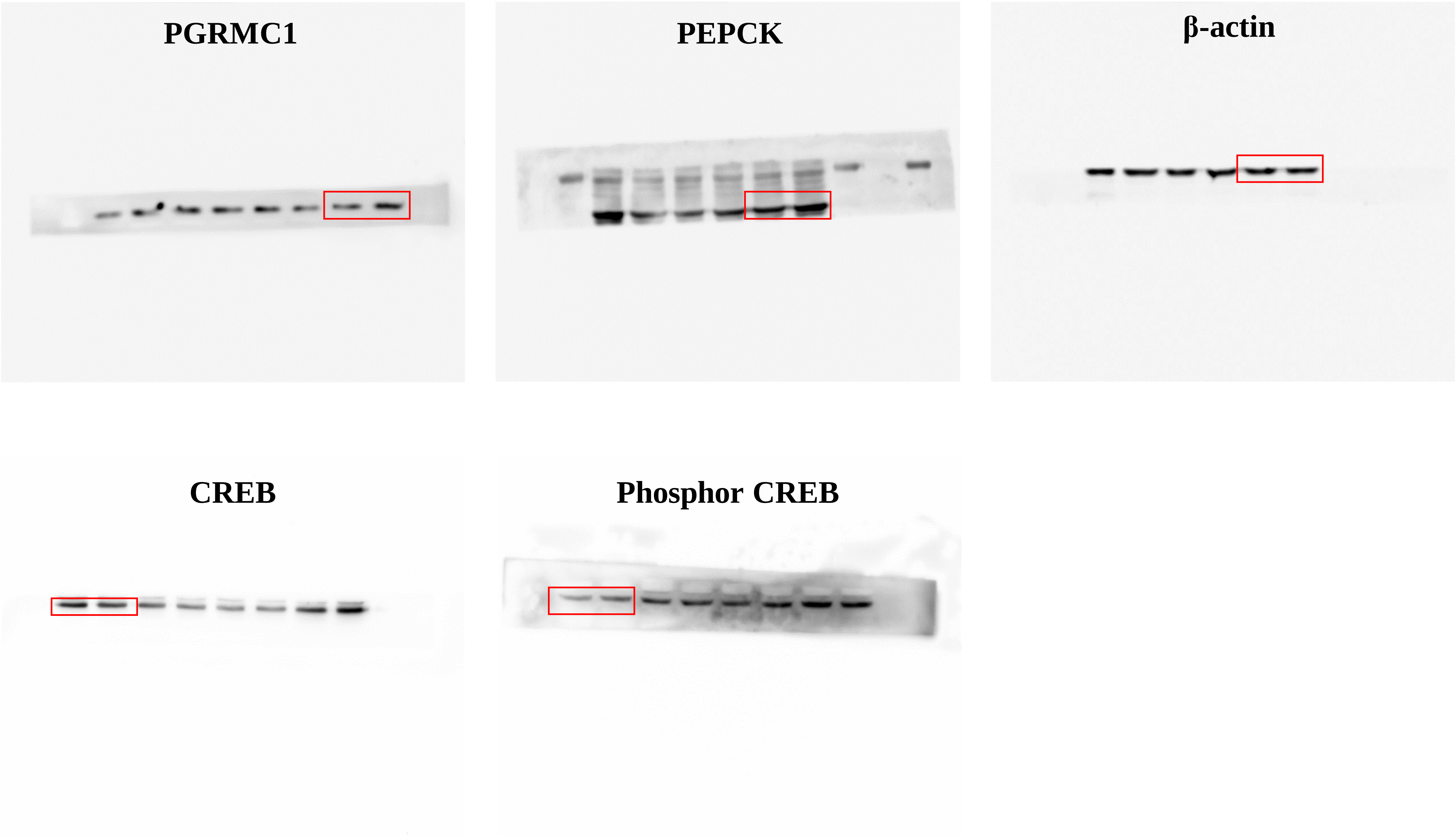

Fig.4C

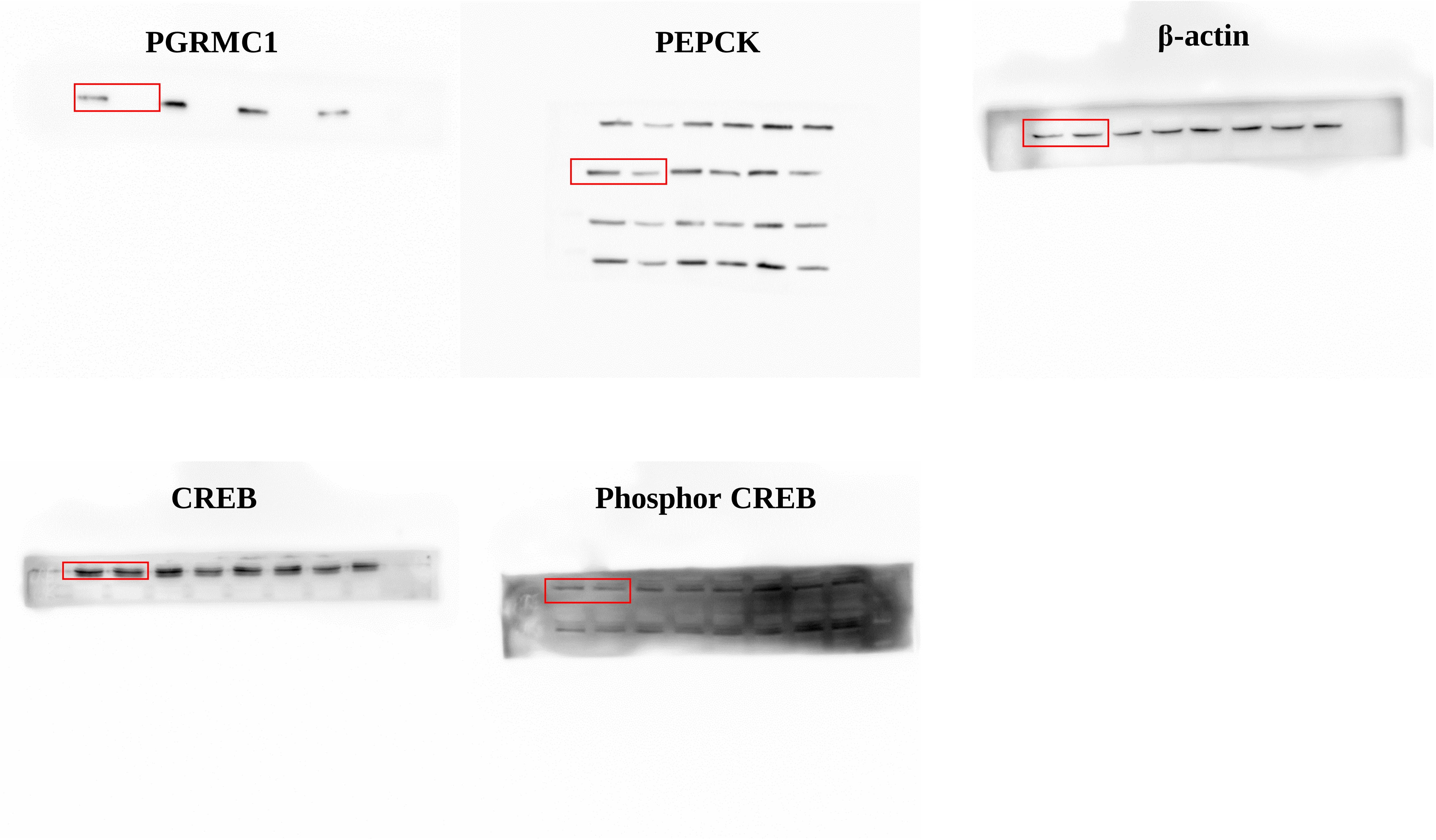

Fig.5A

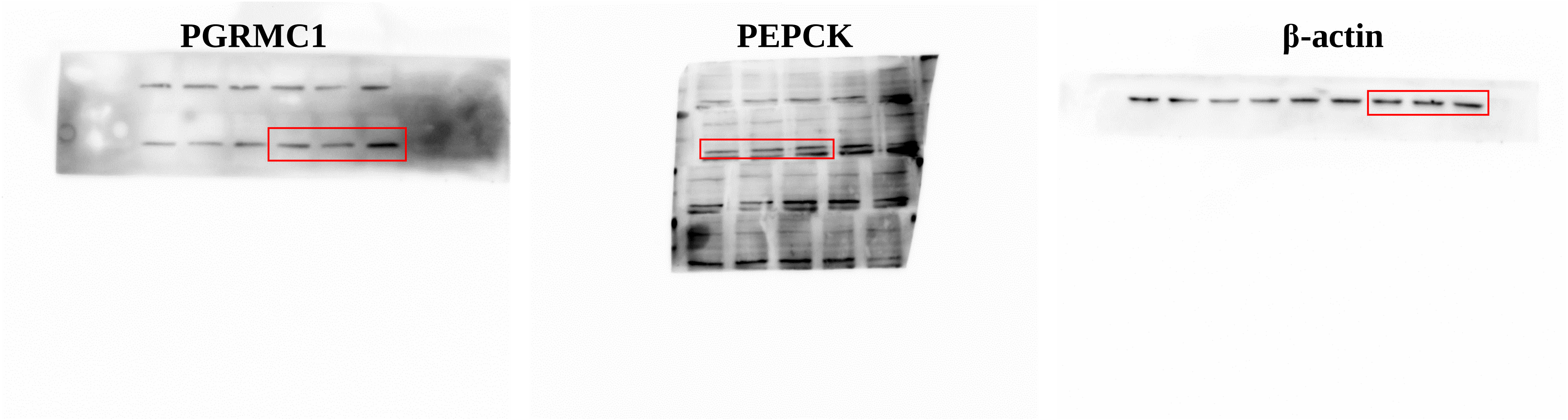

Fig.5C

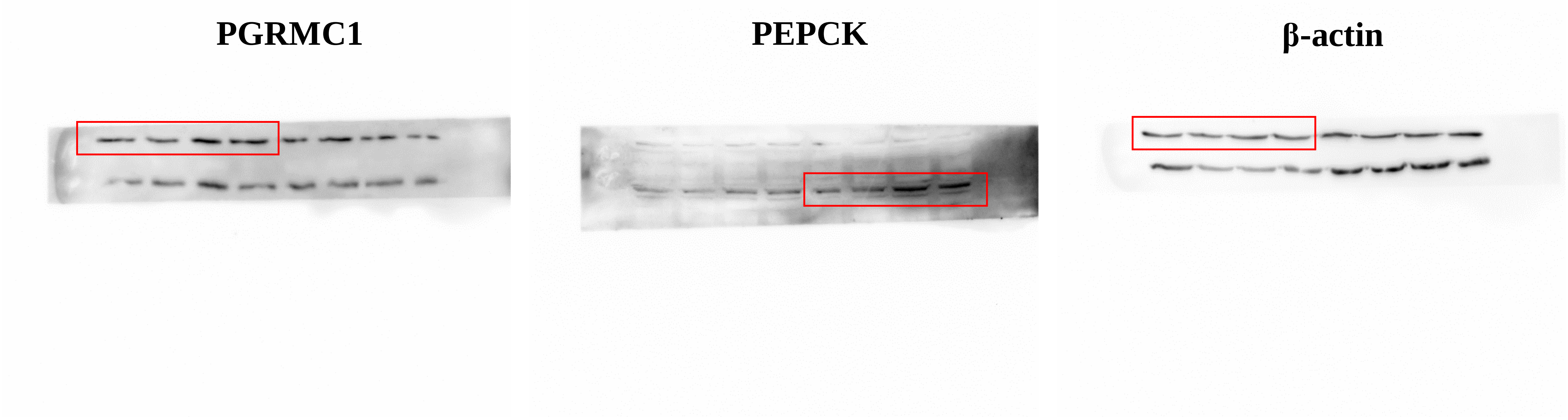

Fig.6A

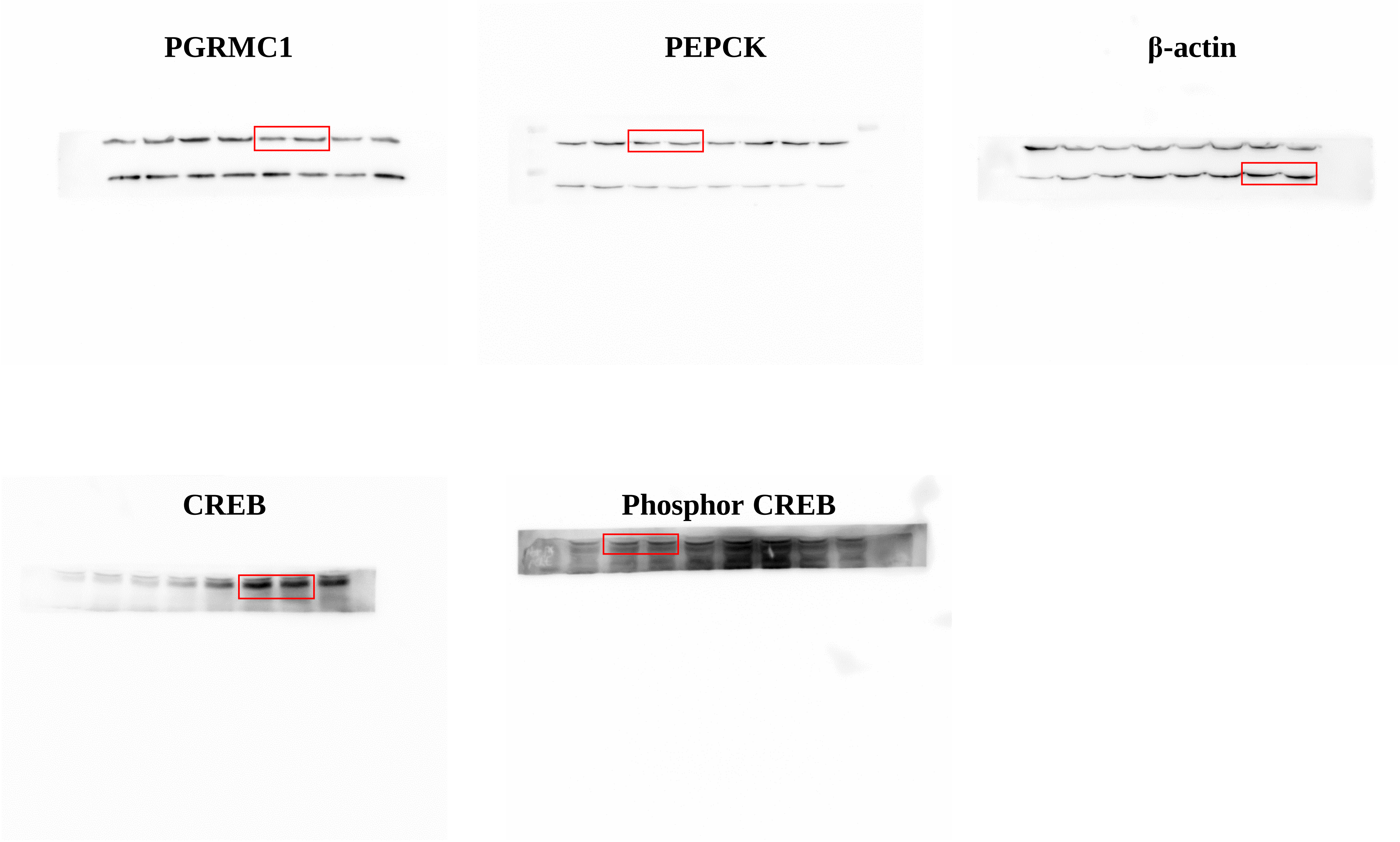

Fig.6B

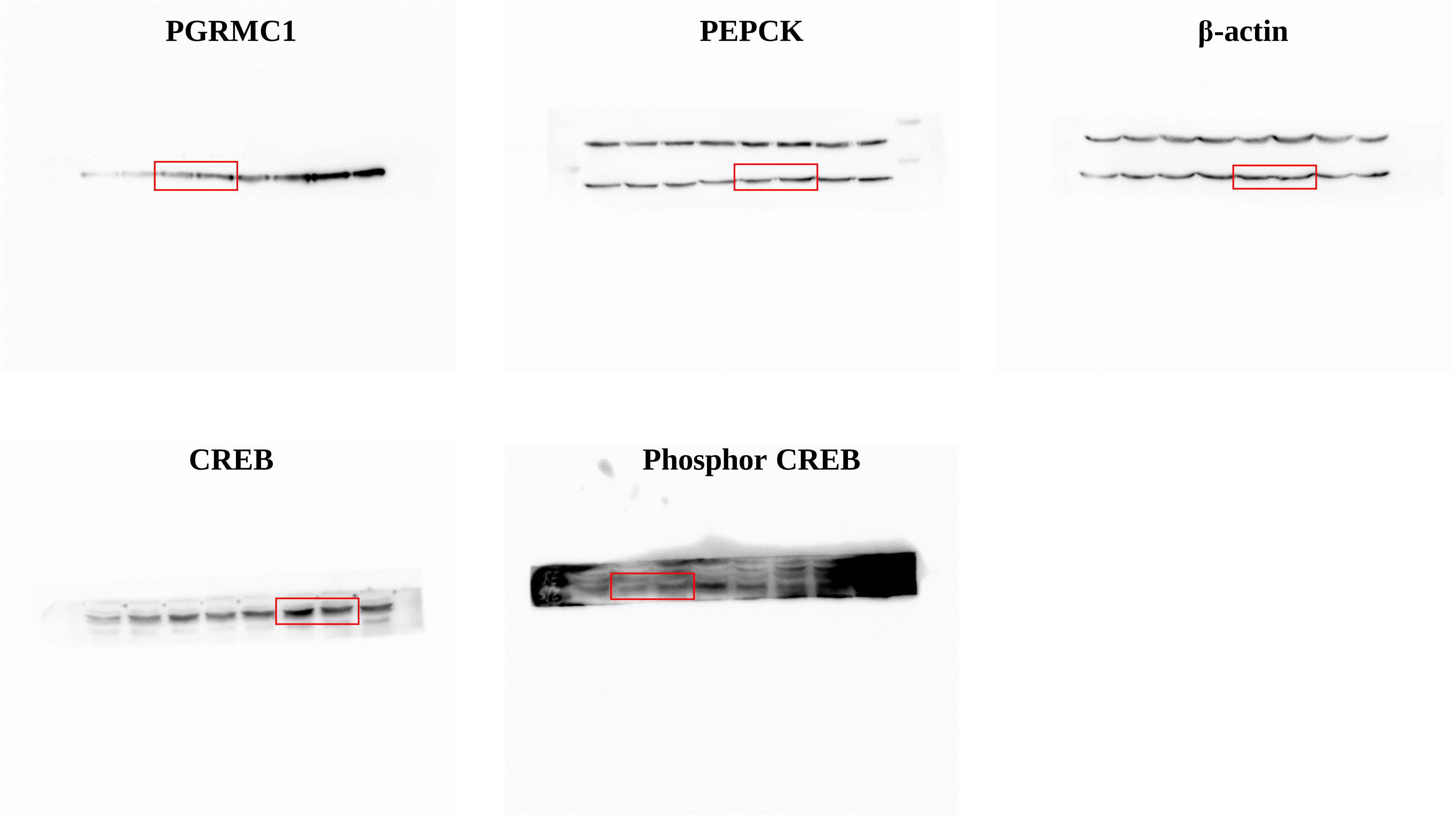

Fig.6C

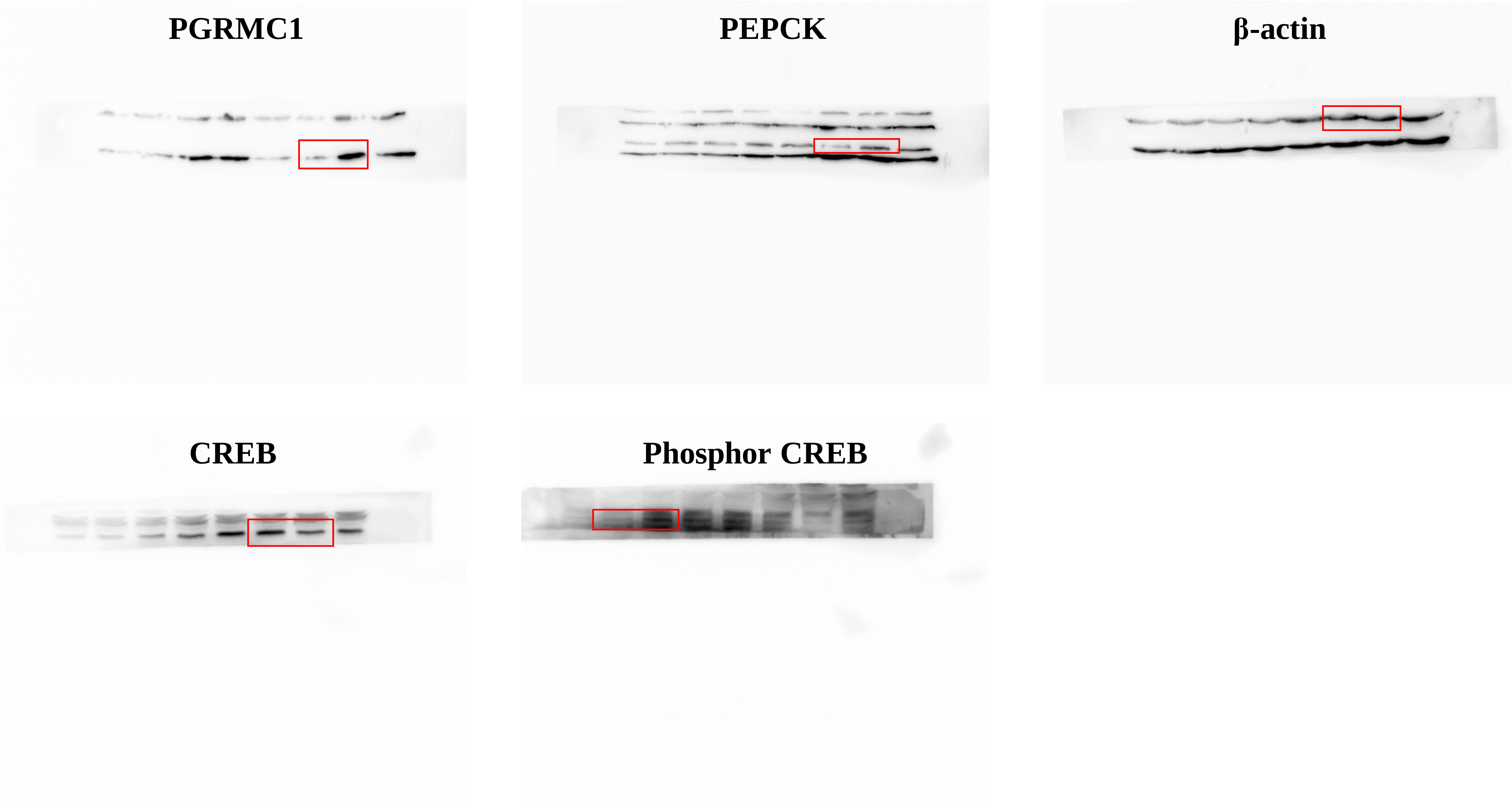

Fig.S2

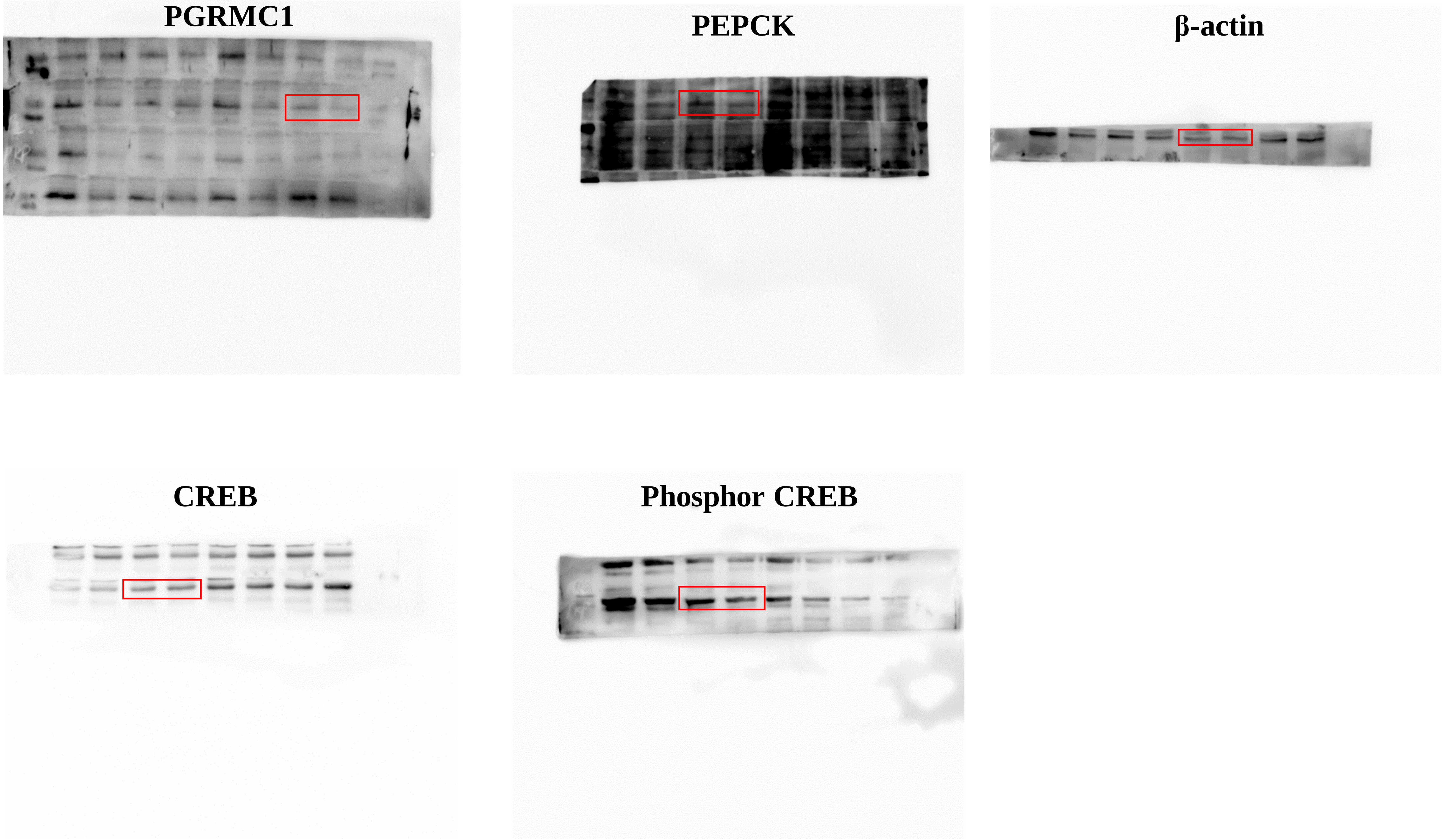

Fig.S4

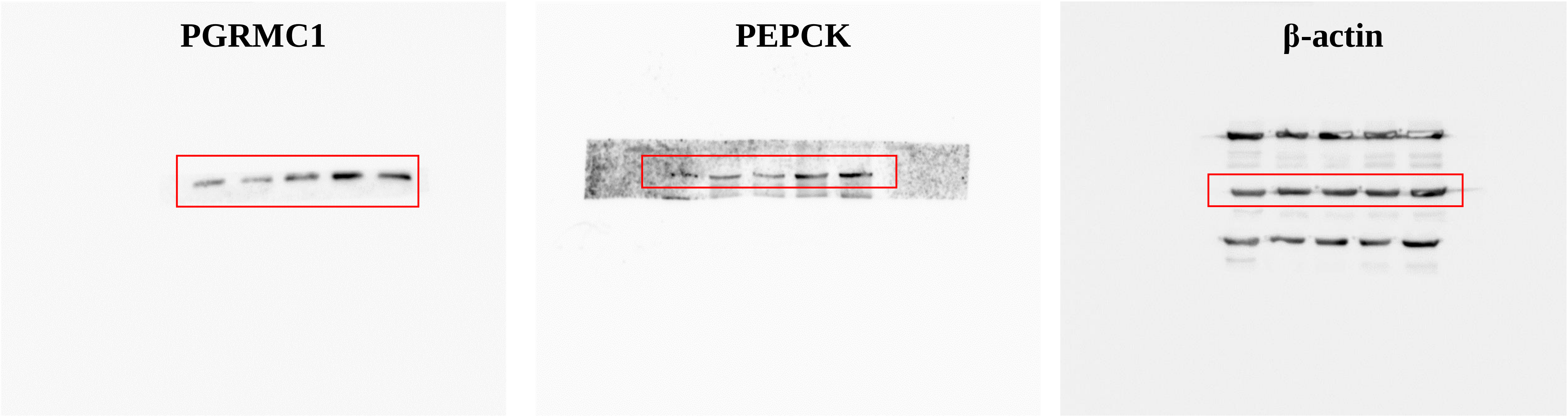

Supplement: Supplementary file 1 — Supplementary Information. [file 41598_2020_73330_MOESM1_ESM.pdf]
